# Association genetics of bunch weight and its component traits in East African highland banana (*Musa* spp. AAA group)

**DOI:** 10.1007/s00122-019-03425-x

**Published:** 2019-09-16

**Authors:** Moses Nyine, Brigitte Uwimana, Violet Akech, Allan Brown, Rodomiro Ortiz, Jaroslav Doležel, Jim Lorenzen, Rony Swennen

**Affiliations:** 1 International Institute of Tropical Agriculture, P.O. Box 7878, Kampala, Uganda; 2 International Institute of Tropical Agriculture c/o Nelson Mandela African Institution of Science and Technology, P.O. Box 447, Arusha, Tanzania; 3 Department of Plant Breeding, Swedish University of Agricultural Sciences, P.O. Box 101, 23053 Alnarp, Sweden; 4 Institute of Experimental Botany, Centre of the Region Haná for Biotechnological and Agricultural Research, 78371 Olomouc, Czech Republic; 5 Laboratory of Tropical Crop Improvement, Division of Crop Biotechnics, Katholieke Universiteit, 3001 Leuven, Belgium; 6 Bioversity International, 3001 Leuven, Belgium; 7 Present Address: Department of Plant Pathology, Kansas State University, Manhattan, KS 66506 USA; 8 Present Address: Bill and Melinda Gates Foundation, Seattle, 23350 USA

## Abstract

**Key message:**

The major quantitative trait loci associated with bunch weight and its component traits in the East African highland banana-breeding population are located on chromosome 3.

**Abstract:**

Bunch weight increase is one of the major objectives of banana improvement programs, but little is known about the loci controlling bunch weight and its component traits. Here we report for the first time some genomic loci associated with bunch weight and its component traits in banana as revealed through a genome-wide association study. A banana-breeding population of 307 genotypes varying in ploidy was phenotyped in three locations under different environmental conditions, and data were collected on bunch weight, number of hands and fruits; fruit length and circumference; and diameter of both fruit and pulp for three crop cycles. The population was genotyped with genotyping by sequencing and 27,178 single nucleotide polymorphisms (SNPs) were generated. The association between SNPs and the best linear unbiased predictors of traits was performed with TASSEL v5 using a mixed linear model accounting for population structure and kinship. Using Bonferroni correction, false discovery rate, and long-range linkage disequilibrium (LD), 25 genomic loci were identified with significant SNPs and most were localized on chromosome 3. Most SNPs were located in genes encoding uncharacterized and hypothetical proteins, but some mapped to transcription factors and genes involved in cell cycle regulation. Inter-chromosomal LD of SNPs was present in the population, but none of the SNPs were significantly associated with the traits. The clustering of significant SNPs on chromosome 3 supported our hypothesis that fruit filling in this population was under control of a few quantitative trait loci with major effects.

**Electronic supplementary material:**

The online version of this article (10.1007/s00122-019-03425-x) contains supplementary material, which is available to authorized users.

## Introduction

The ability of banana to produce seedless fruits was the key to banana domestication along with other attributes such as plant vigour, which was a consequence of polyploidization (Simmonds 1962; Heslop-Harrison and Schwarzacher 2007; Cenci et al. 2019). Deciphering the genetic basis of expressed phenotypes is important for breeders to make useful gene introgression that lead to high-yielding and resilient crops. Yield in banana is evaluated by measuring bunch weight per unit area and over a defined time, often 1 year. The components of bunch weight include a number of traits such as number of hands and fruits; fruit length and circumference; and the diameter of both fruit and pulp (Nyine et al. 2017). Fruit filling refers to the sink capacity of the fruit that is directly proportional to the pulp content, or the edible part of banana. Fruit circumference and length indicate how well the banana fruits are filled with pulp. Fruit filling is one of the main traits that banana breeders use in the preliminary selection of hybrids because it contributes significantly to the bunch weight. Simmonds (1962) reported that parthenocarpy in banana was controlled by three genes designated as *P*_1_, *P*_2_ and *P*_3_, while Turner and Gibbs (2018) described the process of bunch formation and asserted that photosynthate availability plays an important role in regulating the number of fruits and hands on the developing banana inflorescence. There is still, however, a knowledge gap concerning the genetic factors regulating bunch component traits in this crop with important consequences to both food security and economic development in emerging markets.

In well-investigated monocots such as wheat, barley and rice, grain yield has been shown to be controlled by a network of genes involved in plant height determination, flowering regulation, floral architecture, and grain number, width and length (Nadolska-Orczyk et al. 2017; Dixon et al. 2018; Zhang et al. 2018). The available literature shows that in *Musa acuminata, MCM1*-*AGAMOUS*-*DEFENSINS*-*SRF* (MaMADS) genes, MYB and AP2/ERF transcription factors are involved in fruit architecture, development and ripening (D’Hont et al. 2012; Liu et al. 2017). In plantain hybrids, variation in fruit size and parthenocarpy were linked to the segregation of one dominant parthenocarpy gene *P*_1_ (Ortiz and Vuylsteke 1995). Moreover, in triploid banana clones (AAA genomes), three dominant *P* genes have been reported to control parthenocarpy. A parthenocarpic diploid clone Pisang Lilin (AA genome), was shown to be heterozygous for all the three *P* genes while variation in the number of dominant alleles has been reported in other diploids (Simmonds 1962). In spite of the available genomic resources, the location and mode of action of these *P* genes are not fully understood.

Wild diploid progenitors of cultivated banana are the main sources of host plant resistance to pathogens and pests. They have, however, inferior fruit characteristics that are often inherited together through linkage drag with the resistance genes during the breeding process. The consequence of this is that hybrids from breeding programs are sometimes less acceptable by the farmers due to substandard fruit qualities when compared to the existing cultivars (Ortiz et al. 1995). Production of seedless fruit by hybrids arising from non-parthenocarpic and parthenocarpic parents is not unusual (Swennen and Vuylsteke 1993; Vuylsteke et al. 1993a, b), but the majority contain very little, or no pulp. The lack of pulp (poor fruit filling), is one of the main reasons why approximately 90% of hybrids generated in crossbreeding programs are discarded by breeders in early evaluation trials. Early identification of hybrids with poor fruit filling characteristics will save considerable breeding resources. Research by Sardos et al. (2016) noted several loci associated with the seedless phenotype in a panel of 105 diploids, but the disconnection between seedlessness and fruit filling in triploid breeding populations raises an important question, whether the quantitative trait loci (QTL) controlling seedless fruit production are the same as those responsible for fruit filling. Simmonds (1953) pointed out that there was no absolute relationship between seed sterility and parthenocarpy in bananas. In rice, a large effect QTL on chromosome 7 was associated with grain width and length and was complemented by a QTL with smaller effect on chromosome 2 (Begum et al. 2015). Grain weight in wheat is controlled by the *TaGW2* gene, an orthologue of the rice gene *OsGW* involved in grain development. It is known to be a negative regulator of grain weight by modulating cell number and length (Zhang et al. 2018). Low expression of this gene results in increased seed width and reduction in seed length, but generally increases the thousand kernel weight. Perhaps, similar mechanisms are involved in the control of banana fruit size, but such genome-wide association studies (GWAS) or association genetics research have not been attempted in banana.

GWAS relies on high-density molecular markers such as single nucleotide polymorphisms (SNPs) to detect historical and present recombination events mostly in diversity panels (Huang and Han 2014; Begum et al. 2015; Sardos et al. 2016). This makes GWAS a useful tool for identifying genomic loci that are associated with traits of interest (Platt et al. 2010). The number of potentially useful SNPs available for analysis in GWAS is dependent on the genotyping platform, and size and genetic diversity of the utilized population. SNPs can be increased by imputation from a densely genotyped reference panel into a low-density genotyped target population provided identity by descent exists between them (Browning and Browning 2016). SNPs arise from mutations that occur in the life history of a species and the persistence of a certain pattern in a population in disequilibrium to a given trait can be detected through GWAS. However, care must be taken to ensure that genetic heterogeneity due to population structure, relatedness, variation in phenotyping and genotype ascertainment bias do not confound the GWAS results if they are to be reproducible (McCarthy et al. 2008).

The power of GWAS depends on the ability to detect non-random association of SNP alleles linked to the genes that control the trait of interest. This is commonly referred to as linkage disequilibrium (LD). LD has been utilized in human GWAS to identify causal alleles for various diseases (Kerem et al. 1989; Corder et al. 1994) and in plants to identify QTL that control important agronomic traits including yield, or yield components (Buckler et al. 2009; Peiffer et al. 2014; Begum et al. 2015; Sardos et al. 2016). Changes in LD (LD decay) are influenced by the mating design in the population, the frequency of recombination, the distribution of recombination hotspots and population structure (Flint-Garcia et al. 2003; Dyer et al. 2007; Sabeti et al. 2007). LD decay has been shown to vary widely among species and even between subpopulations of the same species and can range from a few hundred base pairs to several hundred kilobase pairs (Flint-Garcia et al. 2003). In a diploid banana diversity panel, rapid LD decay was observed between 10 and 100 kb, but long-range LD was also noted (Sardos et al. 2016).

Inter-chromosomal LD also referred to as genome-wide LD is a phenomenon that reflects the complex control of traits through several regulatory pathways and epistasis. For this reason, Boyle et al. (2017) proposed an ‘omnigenic’ model to account for the effect of modifier genes outside the core pathways involved in the expression of complex phenotypes. Although the use of this concept of inter-chromosomal LD is not widespread in GWAS, careful examination of this concept could uncover the mystery of “missing heritability” in GWAS (Manolio et al. 2009). Indeed, some research articles indicate the existence of non-homologous LD in animals and plants (Farnir et al. 2000; Rostoks et al. 2006; Kulminski, 2011). Using long-range and inter-chromosomal LD, SNPs whose probability (*P*) value is below the threshold of significant association with the trait could be identified. In humans, this concept has been exploited to highlight the role of autophagy in Crohn’s disease, the role of adipocyte thermogenesis and central nervous genes in obesity (Jostins et al. 2012; Claussnitzer et al. 2015; Locke et al. 2015).

In this study, we sought to understand the genetics underlying bunch weight and its component traits in banana through GWAS. Following the high predictive ability of fruit filling traits (> 0.7), in the genomic selection training population (Nyine et al. 2018), we hypothesized that fruit filling was under control of a few QTL with major effects. The objectives were to identify the genomic loci associated with bunch weight and its component traits, the genes closest to, or containing significantly associated SNPs, the level of LD that exists between and within loci associated with the traits and how fast LD decays along the chromosomes having significant marker–trait associations in comparison to the rest of the chromosomes. We also wanted to find out if inter-chromosomal LD exists in banana and to what level could it be utilized to identify the QTL for complex traits such as bunch weight.

## Materials and methods

### Breeding population

The banana population used in this study was described in detail by Nyine et al. (2017) and Nyine et al. (2018). In brief, the population consisted of 307 genotypes from an East African highland banana (EAHB) breeding program of the International Institute of Tropical Agriculture (IITA, Namulonge, Uganda). The population was made up of 31 breeding clones (11 diploids, 12 triploids and 8 tetraploids), and 276 hybrid offspring most of which were triploids. Three fields were established using a completely randomized design with three replications per genotype. Two fields were established at Namulonge in central Uganda, at 0.53°N 32.58°E, while the third field was established in Western Uganda at the Mbarara Zonal Agricultural Research Development Institute, 0.60°S, 30.58°E. One field at Namulonge was maintained under low input management (no mulching and no application of synthetic fertilizer), while the second field and the one at Mbarara were maintained under high input management, which included mulching and addition of 480 g of nitrogen, phosphorus and potassium (NPK), fertilizer per mat per year mixed in the ratio of 25:5:5. Data for three crop cycles were collected from the three fields in the period between 2013 and 2018 because the field in Mbarara was established in 2015, and generally, bananas do not have synchronized flowering and harvesting period.

### Phenotype data

Bunch weight component traits data used in the study included the number of hands and fruits; fruit length and circumference; and diameter of both fruit and pulp. Detailed description of these traits is documented by Nyine et al. (2017). A linear mixed-effect model with restricted maximum likelihood implemented in R-package lme4 (R Core Team 2019) was used to fit the phenotype data as follows,

$$ {y_{ijk}}\; = \;\mu \; + {G_i}\; + {E_j}\; + {C_k} + {\text{GE}}{{\text{C}}_{ijk}}\; + \;{\varepsilon_{ij}} $$

In the equation, *y*_*ijk*_ is the phenotype of the *i*th genotype in *j*th field and *k*th crop cycle, *μ* is the intercept, *G*_*i*_ is the *i*th genotype, *E*_*j*_ is the *j*th field, *C*_*k*_ is the *k*th cycle, GEC_*ijk*_ is the genotype by field by crop cycle interaction and *ε*_*ij*_ is random residual. The effect of field was fixed, while the effects of cycle and genotype were random. The variance components from the mixed-effect linear model were used to calculate the broad sense heritability (*H*^2^) of the bunch weight and its component traits using the equation below.

$$ {H^2}\; = \;\frac{{{V_{\text{G}}}}}{{{V_{\text{G}}}\; + \frac{{{V_{\text{C}}}}}{3}\; + \;\frac{{{V_{\text{GEC}}}}}{9}\; + \;\frac{V_\varepsilon }{27}\;}} $$

where *V*_G_ is the variance of the genotype, *V*_C_ is the variance of the crop cycle, *V*_GEC_ is the variance of the genotype interaction with the field and crop cycle and ${V_\varepsilon }$ is the variance of random residual. Best linear unbiased predictors (BLUPs) were extracted from the model as random effects of the genotype and used as pseudo-phenotypes for GWAS.

### Genotype data

The population was genotyped using the genotyping by sequencing (GBS) approach (Elshire et al. 2011) and ensuing data were processed as described by Nyine et al. (2018). Raw vcf files for the SNP data can be accessed from ftp://ftp.musabase.org/musaGBS/Nyines2018/. Raw SNPs with minor allele frequency (MAF) below 0.05 and maximum missingness above 75% were filtered out using vcf-filter tools, which resulted in 27,178 bi-allelic SNP sites based on double-haploid Pahang genome sequence assembly v2 (Martin et al. 2016). The missing SNPs were imputed using Beagle software program v5.0 (Browning and Browning 2016). A custom Perl script was used to convert the SNP vcf file to haplotype map (hmp) format required for downstream analysis based on IUPAC nucleotide nomenclature.

### Population structure and kinship

The SNP hmp file was imported in TASSEL v5 (Bradbury et al. 2007) and the first three principal components (PCs) were calculated. Genotypes were plotted on the first two PCs in order to delineate the population structure using R-package ggplot2. Hybrid genotypes and the parental lines were identified using different colours on the plot and ellipses were used to identify individuals belonging to the same group by assuming a multivariate normal distribution. Similarly, the kinship coefficients were calculated from the SNP data and used together with the first three PCs to correct for any possible confounding of trait–marker association due to population structure and relatedness.

### Linkage disequilibrium (LD)

In order to determine the number of possible loci that control bunch weight and its component traits in banana, pairwise LD between SNPs were determined with the squared coefficient of correlations between alleles (*r*^2^) and the standardized disequilibrium parameter (*D*′) calculated in TASSEL v5 using an LD sliding window size of 50. A threshold *r*^2^ value was set at 0.1, below which SNP pairs were considered to have weak LD. All SNPs that had a significant association with bunch weight and its component traits were compared with the rest of the SNPs. SNPs that had LD equal, or above 0.1 with the significant SNPs were considered to be associated with the trait even though their *P* values were above the statistically significant threshold. Intergenic SNPs within 1 to 10 kb window were considered to be on the same locus. LD decay plots of *r*^2^ against the physical distance in base pairs (bp) between SNPs from all chromosomes and from individual chromosomes were generated using R-package ggplot2. A smoothened LD curve generated from generalized additive model (GAM) implemented by geom_smooth option of ggplot2 was added to the LD plot for proper visualization of the LD decay trend. Inter-chromosomal LD was searched in the LD data to determine whether some SNPs had an *r*^2^ equal, or above 0.1 with SNPs significantly associated with the traits. The number of SNPs with *r*^2^ equal, or above 0.1 was recorded together with the *D*′ values for each chromosome pair.

### Genome-wide association study

The mixed linear model (MLM) implemented in TASSEL v5 including both kinship matrix (K) and the first three PCs to account for population structure (PCA) was used to test for the marker–trait association. BLUPs were used as pseudo-phenotypes for bunch weight and its component traits. The quartile–quartile (Q–Q) plots for the negative log10-transformed expected and observed *P* values for the SNPs were generated for each trait using R-package ‘qqman’ (R Core Team 2019). These were used to visualize any deviance from the null hypothesis of no genetic association between SNPs and the trait. Manhattan plots for the negative log10-transformed raw *P* values and false discovery rate (FDR) adjusted *P* values against each chromosome were also generated for each trait using R-package qqman. Bonferroni correction was applied to the raw *P* values at 5% significance level (*α* = 0.05), and the threshold *P* value was 1.84e^−6^ (0.05 divided by 27,178 SNPs), for the SNPs that were significantly associated with the traits. The results from Bonferroni correction were compared to those of FDR at a threshold *q* value of 0.05. In order to eliminate any biases due to ploidy level effect, the diploids and tetraploids were excluded from the data set and GWAS was performed only on the triploids since they constituted more than two-thirds of the entire population and results were compared to those of the whole population based on FDR *q* values. Lastly, the level of stringency was reduced to a threshold *q* value of 0.1 so that more SNPs potentially associated with bunch weight and its component traits could be identified.

### Linking significant SNP to putative genes

The gff3 file for double-haploid Pahang genome sequence assembly v2 (Martin et al. 2016) was downloaded from https://banana-genome-hub.southgreen.fr/. Using a custom Perl script, bed files were generated for the gene models from gff3 and the SNPs that were significantly associated with bunch weight and its component traits. The closest-features option from BEDOPS v2.4.35 tools (Neph et al. 2012), was used to determine the gene that contained, or was closest to the significant SNP. The annotation of the gene was obtained from the gff3 file using the gene identifier. To further gain a deeper understanding, the effects of all SNPs used in this study were annotated using SnpEff software (Cingolani et al. 2012). The database for double-haploid Pahang genome sequence assembly v2 was locally built in SnpEff software following the user manual before the annotation was done because the default settings could only allow access to the first version of the *Musa* genome present at Ensembl genomes website.

## Results

### Heritability and population structure

After filtering the data for missing entries and obvious scoring errors, 297 accessions were retained that had data from all the three experimental fields. These accessions were used to calculate the variance components of the variables, BLUPs and other downstream analyses (Supplementary material Table S1). The *H*^2^ for bunch weight was 0.92, number of hands was 0.88, number of fruits was 0.83, fruit length was 0.98, fruit circumference was 0.98, fruit diameter was 0.98 and pulp diameter was 0.97.

**Fig. 1:**
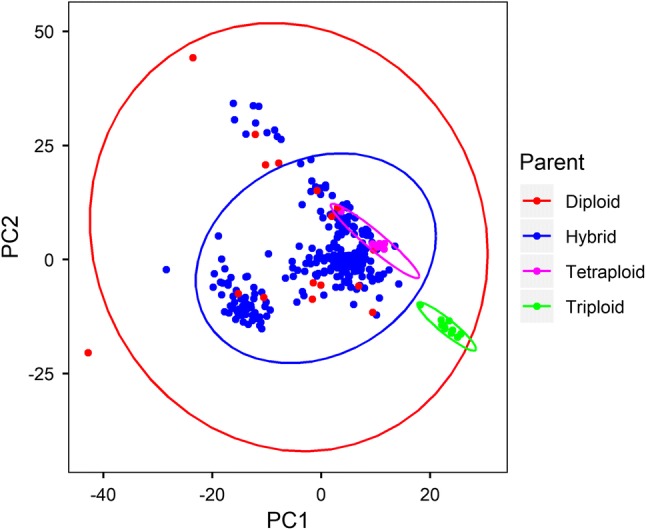
Distribution of genotypes in the study population on the first and second principal components. Where diploid (red), triploid (green) and tetraploid (magenta) are the parents that generated the hybrids (blue) (color figure online)

PCA of these accessions based on 27,178 SNPs did not reveal a clear distinct population structure amongst the hybrids assuming a multivariate normal distribution (Fig. 1). However, they showed a wide distribution on the two PCs, which was indicative of the high genetic variability despite some shared pedigree. Among the parents, the triploid EAHB landraces formed a unique cluster away from the rest of the population, while the tetraploids clustered together amongst the hybrids. The diploid parents had the widest spread on the two PCs (Fig. 1).

### Linkage disequilibrium

**Fig. 2:**
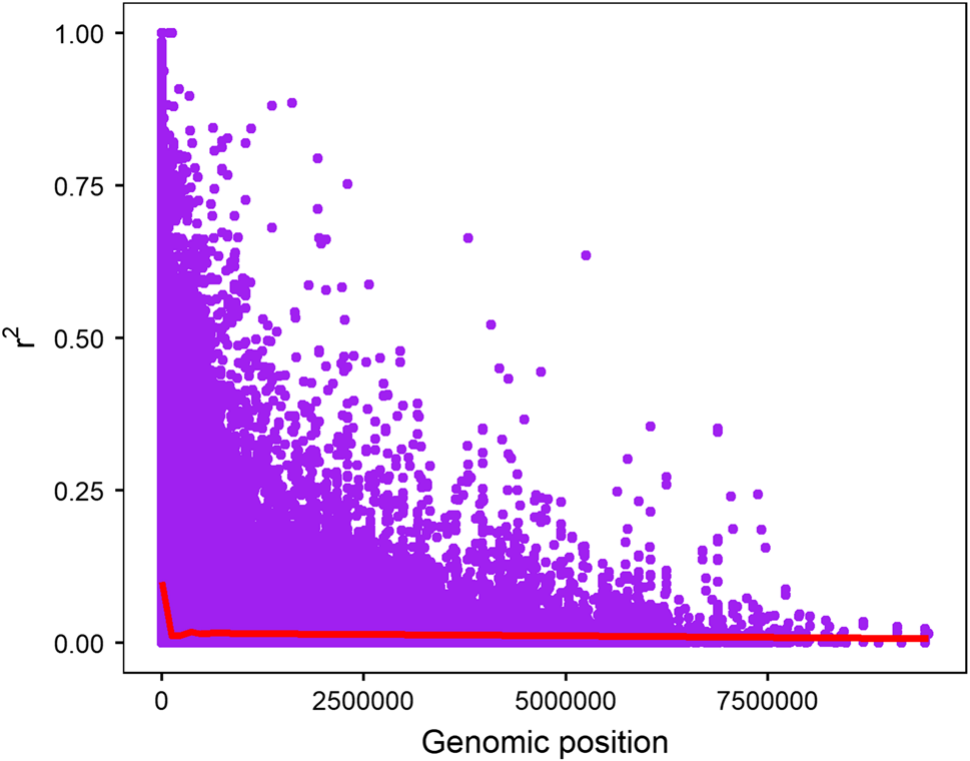
Linkage disequilibrium plot of *r*^2^ against genomic distance between all SNP pairs. The smoothened curve (red) was generated using generalized additive model implemented in gglpot2 geom_smooth function (color figure online)

Pairwise analysis of LD between 27,178 SNPs in TASSEL v5 produced 1,357,625 comparisons. The number of chromosome-specific pairwise SNP LD was 1,287,876 out of which only 4.6% (59,263) had *r*^2^ equal, or above 0.1. The remaining pairs were from inter-chromosomal LD and unanchored scaffolds (labelled as chromosome U). The average *r*^2^ and *D*′ for the 4.6% pairs were 0.29 ± 0.26 and 0.73 ± 0.22, respectively. Inter-chromosomal LD SNP pairs between the 11 chromosomes were 11,281 out of which only 0.5% (56) had *r*^2^ equal, or above 0.1. The average *r*^2^ and *D*′ for the 0.5% pairs were 0.15 ± 0.04 and 0.53 ± 0.14, respectively. The smoothened LD curve from GAM for all SNP pairs showed a very sharp drop in *r*^2^ within the first 10 kb followed by a slight increase before stabilizing (Fig. 2). The average *r*^2^ for the first 100 kb was 0.38 ± 0.31. However, long-range LD with *r*^2^ equal, or above 0.1 was observed across all chromosomes and it varied from 1.9 Mb on chromosome 11 to 7.5 Mb on chromosome 7 (Table 1). Only 0.72% of SNP pairs had long-range LD with *r*^2^ equal, or above 0.5 at a distance ranging between 0.9 Mb on chromosome 7 and 4.1 Mb on chromosome 5 (Table 1). Chromosome 3 had a unique trend in the LD smoothened curve because after 1.5 Mb, it started to show an increase in *r*^2^, while other chromosomes remained constant, or slightly reduced (Supplementary material Fig. S1). The average LD distance (and standard error) between SNPs significantly associated with bunch weight and its component traits based on the entire population and triploid subset was 0.97 ± 0.1 Mb (1 bp to 2.8 Mb), while the average *r*^2^ was 0.28 ± 0.03 (0.003 to 1) and *D*′ was 0.63 ± 0.03 (0.16 to 1).

**Table 1:** Distribution of pairwise SNP linkage disequilibrium (LD) amongst the eleven chromosomes within the banana-breeding population at a threshold *r*^2^ equal, or above 0.1 and *r*^2^ equal, or above 0.5

Chrom	Pairwise SNP LD			Percentage		Long-range LD	
	Total pairs	*r*^2^ ≥ 0.1	*r*^2^ ≥ 0.5	0.1 (%)	0.5 (%)	0.1 (Mb)	0.5 (Mb)
Chr1	97,120	4709	733	4.85	0.76	3.2	1.0
Chr2	78,223	3951	624	5.05	0.80	4.6	1.7
Chr3	124,863	5974	941	4.78	0.75	2.8	2.3
Chr4	141,991	6394	1057	4.50	0.74	4.3	1.0
Chr5	114,298	5592	779	4.89	0.68	6.1	4.1
Chr6	156,514	7099	1166	4.54	0.75	3	2.6
Chr7	117,412	5302	823	4.52	0.70	7.5	0.9
Chr8	123,472	5227	887	4.23	0.72	4.5	2.3
Chr9	117,613	5318	911	4.52	0.78	7	2.0
Chr10	115,829	5124	755	4.42	0.65	5.5	3.8
Chr11	100,541	4573	682	4.55	0.68	1.9	1.4

### Marker–trait association

**Fig. 3:**
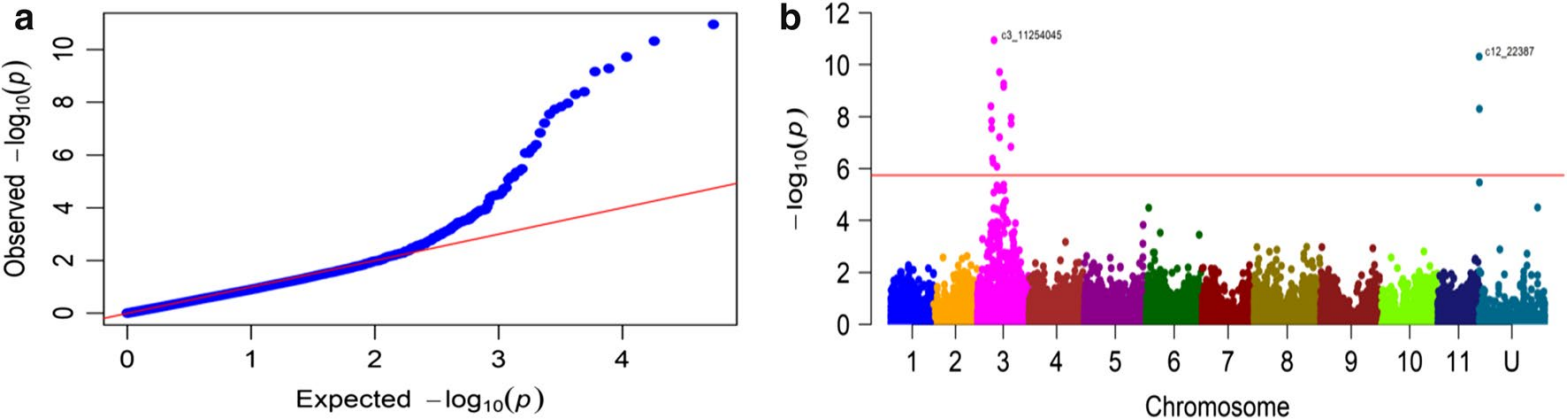
Single nucleotide polymorphisms (SNPs) associated with fruit circumference. **a** Quartile–quartile (Q–Q) plot showing deviation from null hypothesis of no significant association, **b** Manhattan plot showing location of significant SNPs, the red line indicates the Bonferroni correction threshold at a *P* value of 1.84e^−6^ (color figure online)

Mixed linear model accounting for both population structure and kinship, MLM (PCA + K) was used to identify the marker–trait association. The Q–Q plots showed deviation from the null hypothesis, thus indicating the presence of a significant association between SNP markers and bunch weight, and its component traits (Fig. 3a, Supplementary material Fig. S2). To identify the significant SNPs, we used both the Bonferroni correction at a threshold *P* value of 1.84e^−6^ and an FDR at a threshold *q* value of 0.05. All SNPs above the threshold line (red line) on the Manhattan plots were considered to be strongly associated with the traits (Fig. 3b, Supplementary material Fig. S3). Based on Bonferroni correction, 20 SNPs were associated with the number of hands, fruit length and circumference; and the diameter of both fruit and pulp (Table 2, Supplementary material Table S2), and no SNPs were associated with bunch weight and number of fruits. However, based on FDR, 32 SNPs were in strong association with bunch weight, number of hands; fruit length and circumference; and the diameter of both fruit and pulp (Table 2, Supplementary material Fig. S4). Two SNPs on chromosome 10 were weakly associated with the number of fruits based on FDR. Altogether, 34 out of the 27,178 SNPs used in the study, distributed across 20 loci located on five chromosomes and on unanchored scaffolds were significantly associated with bunch weight and its component traits. Twenty-six of the significant SNPs were located on chromosome 3. Chromosomes 6, 9 and 11 had one significant SNP each, while two SNPs were located on chromosome 10 and three SNPs were on unanchored scaffolds (U). The location of SNPs, the putative genes to which they mapped, or that was closest to them and the effect of the SNPs on the traits are summarized in (Supplementary material Table S3). Most of the SNP alleles had a negative effect on the traits.

**Table 2:** List of SNPs significantly associated with bunch weight and its component traits in the whole population (WP) and the triploid subset based on Bonferroni (Bon) correction and false discovery rate (FDR) at 5% significance level

Trait	Chrom	SNP position	WP. Bon	WP. FDR	Tri FDR
BWT	Chr3	6989789	−	+	+
BWT, FL, FC, FRD, PLD	Chr3	9149022	+	+	+
FC, FRD, PLD	Chr3	9149076	−	−	+
FC, FRD, PLD	Chr3	9149101	−	−	+
FC, FRD, PLD	Chr3	9149108	−	−	+
FC, FRD, PLD	Chr3	9754001	+	+	+
FC, FRD, PLD	Chr3	9754011	+	+	+
FC, FRD, PLD	Chr3	10305590	+	+	−
FL, FC, FRD, PLD	Chr3	10692040	+	+	+
FL, FRD, PLD	Chr3	11198135	−	+	+
FL, FC, FRD, PLD	Chr3	11198169	−	+	+
BWT, FL, FC, FRD, PLD	Chr3	11254045	+	+	+
FC, FRD, PLD	Chr3	13115782	−	−	+
FC, FRD, PLD	Chr3	13115793	−	−	+
FC, FRD	Chr3	13346953	−	+	−
FL, FC, FRD, PLD	Chr3	13347035	+	+	−
FL, FC, FRD, PLD	Chr3	13347036	+	+	−
FC, FRD, PLD	Chr3	14800401	−	−	+
FRD	Chr3	14800413	−	−	+
PLD	Chr3	14800497	−	−	+
FL, FC, FRD, PLD	Chr3	15110523	+	+	+
FC, FRD, PLD	Chr3	15110536	−	+	+
FL, FC, FRD, PLD	Chr3	15110553	+	+	+
FC, FRD*, PLD	Chr3	17545341	−	+	+*
FC, FRD, PLD	Chr3	17545360	−	−	+
FC, FRD, PLD	Chr3	17545361	−	−	+
FC, FRD, PLD	Chr3	17545362	−	−	+
FC, FRD, PLD	Chr3	17857961	+	+	+
FL, FC, FRD, PLD	Chr3	17857966	+	+	+
FC, FRD, PLD	Chr3	17858009	+	+	+
FC, FRD, PLD	Chr3	17908329	−	+	+
FRD	Chr3	18217480	−	+	−
FRD, PLD	Chr3	19044984	−	+	+
FC, FRD, PLD	Chr3	22602825	+	+	+
FL, FC, FRD, PLD	Chr3	22755473	+	+	+
FL, FC, FRD, PLD	Chr3	22755506	+	+	+
FRD, PLD	Chr3	24538797	−	−	+
FRD, PLD	Chr3	24838773	−	−	+
BW	Chr3	29247703	−	+	−
FC	Chr6	1758900	−	+	−
NH	Chr9	35224995	+	+	−
NF	Chr10	32429267	−	+	+
NF	Chr10	32429274	−	+	+
FL	Chr11	25332499	−	+	−
FL, FC, FRD, PLD	ChrU	22387	+	+	+
FL, FC, FRD, PLD	ChrU	214150	+	+	+
FC, FRD, PLD	ChrU	214240	+	+	+

Present (+), Absent (−), bunch weight (BWT), number of hands (NH), number of fruits (NF), fruit length (FL), fruit circumference (FC), fruit diameter (FRD), pulp diameter (PLD), triploid (Tri), chromosome (Chrom), *significant association detected only in the triploid subset

Eight out of the 26 significantly associated SNPs on chromosome 3 are located in, or are near the genes that encode either hypothetical or uncharacterized proteins. Two SNPs mapped to MYB-related protein 308-like gene, which belongs to a family of transcription factors involved in various cellular processes including cell development. Three SNPs are located upstream of the transcription factor bHLH49 known to regulate cell elongation and expansion in *Arabidopsis*. Two SNPs mapped to proline-rich protein HaeIII known to be involved in stress response and cell wall development, one SNP mapped to kinesin-like protein while another one mapped to auxilin-like protein, both involved in intracellular transport. The remaining SNPs on chromosome 3 mapped to F-Box protein, pentatricopeptide repeat-containing protein, random slug protein, putative triactylglycerol lipase 2 and muscle M-line assembly protein unc-89-like. One of the three SNPs on unanchored scaffolds mapped to a gene encoding ribokinase, while the other two SNPs are located in a gene encoding 2-aminoethanethiol dioxygenase-like protein.

### GWAS with triploid genotypes

When GWAS was performed with the triploid subset, most of the SNPs identified using the entire population were also identified in this subset at an FDR threshold *q* value of 0.05 (Supplementary material Tables S2, S3). However, some SNPs that showed association in the whole population (WP) were below the threshold yet, 14 new SNPs were identified to be significantly associated with fruit filling traits and these were all located on chromosome 3. One of the 14 SNPs (c3_17545341), however, was significantly associated with fruit circumference and pulp diameter in the WP but in the triploid subset, it was only significantly associated with fruit diameter (Supplementary material Table S2). These SNPs were distributed on six loci with half of them mapped to the genes such as *UPF0481 protein At3g47200*-*like*, *enhancer of polycomb*-*like protein 1* and *TIMELESS*-*interacting protein* known to be involved in DNA replication and repair, and cell cycle regulation. The other half of the SNPs mapped upstream of *ervatamin*-*B*-*like*, *hypothetical protein* and *membrane*-*anchored ubiquitin*-*fold protein 3*-*like* genes (Supplementary material Table S3). Interestingly, some of the 14 SNPs had strong LD with SNPs that had a significant association with bunch weight and its component traits based on WP.

### Inter-chromosomal and long-range LD SNPs

Only 56 SNP pairs had inter-chromosomal LD with *r*^2^ equal, or above 0.1 from all possible chromosome combinations and none of them was in LD with SNPs significantly associated with bunch weight and its component traits. However, 87 nonsignificant SNPs on chromosome 3 were in LD with SNPs significantly associated with bunch weight and its component traits at *r*^2^ equal, or above 0.1 (Supplementary material Table S4). Gene models closest to these SNPs were not different from those of the significant SNPs. The distance between significant SNPs on chromosome 3 varied from 1 bp to 2.9 Mb with some distant SNP pairs showing high LD. One interesting region was between 15,110,500 and 17,864,044 bp where the *r*^2^ values between the two SNP pairs; c3_15110553 and c3_17857961, c3_15110553 and c3_17857966 were 0.42 and 0.41, respectively, despite a 2.75 Mb distance between them. This explains the trend observed from the LD plot of this chromosome where the smoothened curve started to increase at around 15 Mb. SNP c3_15110553 is located in the promoter region of uncharacterized LOC103978321 gene (Ma03_g15320), while c3_17857961 and c3_17857966 are both located in the coding region of hypothetical protein ~ At3g50860 (Ma03_g16130). These three SNPs have two haplotypes TTA and CCG, which show positive and negative effect on fruit filling related traits, respectively (Supplementary material Table S3). When the interval was scanned, 82 genes were identified and a majority of them were either annotated as encoding uncharacterized, or hypothetical proteins (Supplementary material Table S5). Several genes encoding proteins such as AP2-like/ERF, transcription factor bHLH18-like and bHLH10-like, growth regulating factor 6, auxin-responsive protein IAA1, endoglucanase 24-like, CDPK-related kinase 3-like, ervatamin-B-like, E3 ubiquitin-protein ligase SINAT5-like and others were, however, present. Since most of these genes are involved in cell cycle regulation, cell wall development, cell elongation and expansion, signal transduction and transcriptional regulation of other genes, it is likely that differential expression of these genes affects different hybrid genotypes in diverse ways, resulting in the source to sink capacity variation. Using a 1 Mb sliding window, the SNP count in the interval was only 69 barely above the average of 66 SNPs per 1 Mb for the whole genome (Supplementary material Fig. S5). The region between 16 and 17 Mb on chromosome 3 had only 4 out of the 69 SNPs, which limited the possibility to detect any significant associations in the region. The presence of long-range LD suggested, therefore, that this interval bears genes that could affect bunch weight and its component traits.

### Annotation of SNPs

After relaxing the level of stringency to a *q* value of 0.1, 19 new SNPs were identified that were significantly associated with bunch weight and its component traits. Thirteen of the SNPs were located on chromosome 3, while chromosomes 4, 6, 9 and 10 had one SNP each, and two SNPs were located on the unanchored scaffolds (Supplementary material Table S6). However, linking these SNPs to genes did not reveal any new genes but mapped to genes such as *TIMELESS*-*interacting protein* and others that had been already identified.

When the 27,178 SNPs were annotated using SnpEff software, 12,156 SNPs had functional effects (File S1). There were 53.37% (6488) missense SNPs, 45.76% (5562) silent SNPs and 0.87% (106) nonsense SNPs giving a ratio of 1.17, missense/silent mutations. However, when the base changes (SNPs) were analysed, the ratio of transitions/transversions (Ts/Tv) was 1.37 indicating the presence of a high proportion of SNPs that cause amino acid changes in the genes. At high stringency threshold *P* value and *q* value, we identified four missense SNPs on chromosome 3 (Supplementary material Table S6). Three of them (chr03_14800401, chr03_14800413 and chr03_14800497) were located in the *TIMELESS*-*interacting protein* (Ma03_g15020), while one (chr03_11198135) was present in *MYB-related protein 308-like* (Ma03_g14020). These two genes are known to play a role in cell cycle regulation.

## Discussion

### Population structure in breeding population

Association genetics should rely on high-density genetic markers evenly distributed across the genome to identify QTL associated with traits of interest. In this study, we used 27,178 SNP markers with a minimum minor allele frequency of 0.05 to find association with bunch weight and its component traits in a banana-breeding population derived from the triploid EAHB as grandparents. The average marker density was 66 SNPs per 1 Mb window across all 11 chromosomes, but variations along the chromosomes were noted with a reduction towards the centromeric region. We did not find a clear population structure in our population based on PCA especially amongst hybrids (Fig. 1). The triploid EAHBs formed, however, a unique cluster indicating low genetic diversity between them. Kitavi et al. (2016) and Němečková et al. (2018) showed that this subgroup of bananas is genetically uniform and postulated that it arose from single clone with *M. acuminata* ssp. *zebrina* and ssp*. banksii* being the most probable parents. The tetraploids that arose from them also clustered together indicating that they had a high genetic dosage from the EAHB parents (Batte et al. 2019; Brown et al. 2017; Nyine et al. 2017). The lack of a clear population structure within the hybrids can be attributed to their shared complex pedigree. And the wide distribution of hybrids on the two components reflects high diversity among the male parents.

The distribution and the frequency of marker alleles carried by different subpopulations in diversity panels commonly used in most association studies are influenced by evolutionary dynamics such as mutation rates, gene flow, recombination frequency and geographical isolation among others (Zhao et al. 2011). These evolutionary forces can lead to population structure characterized by genetic heterogeneity and cryptic relatedness that confound the marker-trait associations in such panels (Korte and Farlow, 2013). In breeding populations, however, genetic heterogeneity may not be strong due to the influence of the population structure, but relatedness between individuals is usually high, which can lead to false-positive association (Korte and Farlow, 2013). Mixed linear models accounting for both population structure and kinship have been used in GWAS to minimize false-positive association (Yu et al. 2006; Zhao et al. 2007; Kang et al. 2008; Zhang et al. 2010; Müller et al. 2019), thereby leading to identification of candidate genes and associated markers that can be used in marker-assisted selection for traits in both plant and animal breeding programs. This approach was used to identify the mutations in the *dwarf8* gene that controls flowering time and plant height in maize (Thornsberry et al. 2001), and candidate genes that could be involved in seedless fruit production in bananas (Sardos et al. 2016). We used the first three PCA and kinship matrix derived from the SNP markers to correct for any spurious association followed by the stringent Bonferroni and a relatively relaxed FDR correction method. Hence, we expect that the associations detected in our study point to loci involved in the control of bunch weight and its component traits in banana. Further research including knockout or editing of genes having missense SNPs that are significantly associated with bunch weight and its component traits could lead to identifying genetic factors controlling yield in banana and the development of DNA markers that can be used routinely for selection of hybrids.

### Linkage disequilibrium

Association genetic analysis on diversity panels usually captures linkage disequilibrium that has persisted through historical recombination (Flint-Garcia et al. 2003). In breeding population, both historical and present recombination events are captured. Hence, the results from such analysis are likely to have more immediate application in breeding programs than those from diversity panels that require validation (Begum et al. 2015). In this study, we used a breeding population that was composed of many half-sib families derived from related female parents (Kitavi et al. 2016; Němečková et al. 2018), but diverse male parents. We observed a rapid decay in LD within the first 10 kb on each chromosome, which was lower than that observed in the diploid diversity panel for seedless phenotype in banana (Sardos et al. 2016). The trend of LD decay based on the smoothened curves generated by GAM was comparable to that observed by Sardos et al. (2016), where rapid LD decay was followed by long-range LD. Tremendous differences in LD decay were reported in various species. In humans, LD decay was reported to vary from 60 to 500 kb, in other animals it varied from 1 kb to several centimorgans (Remington et al. 2001). In maize, LD reduced to less than 25% within the first 200 bp, yet in the diverse inbred lines of maize, rapid LD decay was observed at 1500 bp (Remington et al. 2001), while in *Arabidopsis thaliana* LD decayed within 250 kb to several megabases depending on the population and gene under selection (Flint-Garcia et al. 2003).

Long-range LD varied from 1.9 to 7.5 Mb in our population. The variation in LD has been attributed to mating designs in population such as outcrossing versus selfing, rate of recombination and the distribution of recombination hotspots, population bottlenecks during domestication, locked genomic portions due to presence of repetitive sequences and continuous selection of domestication genes (Labate et al. 2000; Jeffreys et al. 2001; Rafalski 2002; Weil, 2002; Whitt et al. 2002; Jordan et al. 2018). Unlike highly selfing species such as *Arabidopsis* and wheat, banana can maintain long-range LD blocks due to its vegetative propagation that limits the number of recombination events. Hence, the high LD observed in this study could be an indication of the selection pressure that banana underwent during the domestication process and has been maintained by vegetative propagation. The occurrence of large linkage blocks could be a problem for banana improvement if unfavourable alleles are present in such blocks, but if they contain only favourable alleles, fixation of the traits controlled by those loci in the hybrids becomes easy. The reduction in LD decay range compared to the diversity panel of Sardos et al. (2016), could be due to recent meiotic recombination following cross-pollinations. In *Drosophila recens*, genome-wide LD observed within the driving X chromosome (X^D^) was attributed to the presence of multiple inversions that suppressed recombination of X^D^ with wild type thus generating non-random association between alleles on a 130 cM haplotype (Dyer et al. 2007).

Although we observed some inter-chromosomal LD between SNP markers, none of them had any significant association with bunch weight and its component traits, indicating that this phenomenon may not play a role in bunch weight and its component traits in banana. Kulminski (2011) observed, however, inter-chromosomal LD in both parental and offspring generation of Framingham heart study participants and concluded that it was caused by biogenetic mechanisms possibly associated with favourable, or unfavourable epigenetic evolution. Further research using other populations with different genetic background and traits will be therefore required to rule out the role of inter-chromosomal (genome-wide) LD in banana trait-marker associations.

### Markers associated with bunch weight and its component traits

After accounting for population structure and kinship in the mixed linear model, we identified a total of 34 SNPs using the entire population and an additional 14 SNP using the triploid subset and when combined together, 47 unique SNPs were significantly associated with bunch weight and its component traits (Table 2). Altogether, they mapped to 25 genomic loci located on chromosomes 3, 6, 9, 10 and 11, and on unanchored scaffolds, the majority of which were located in protein-coding regions while a few were in the promoter, or 3′ UTR regions based on their physical distances from the closest putative gene. Most genes having significant SNPs encoded either uncharacterized, or hypothetical proteins based on the double-haploid Pahang reference genome annotation (D’Hont et al. 2012; Martin et al. 2016). Some of the SNPs were, however, located in genes with known functions and these included mostly transcription factors such as MYB-related protein gene, AP2-like/ERF, basic helix-loop-helix-like (bHLH-like) family of transcription factors that were found on chromosome 3. These transcription factors are well characterized in model plants such as *Arabidopsis thaliana* and other plants and were shown to be involved in diverse cellular developmental, differentiation and metabolic processes (Dubos et al. 2010; Feller et al. 2011; Ambawat et al. 2013; Pireyre and Burow, 2015).

Besides transcription factors, other genes that were identified on chromosome 3 through long-range LD SNP included genes that control DNA replication, gene transcription and cell cycle regulation such as *enhancer of low casing for polycomb*-*like protein 1*, *TIMELESS*-*interacting protein*, *CDPK-related protein kinase 3*-*like*, *ervatamin*-*B*-*like* and *E3 ubiquitin*-*protein ligase SINAT5*-*like* amongst others (Levine et al. 2004; Borde & Lichten 2014). *Enhancer of polycomb proteins* were shown to be involved in epigenetic maintenance of gene expression patterns and posttranscriptional modification in cells (Levine et al. 2004). In *Nicotiana tabacum*, gene *NtCDPK1* was shown to be mainly expressed in proliferating root and shoot meristems as well as in developing floral buds. Inhibition of its expression was associated with abnormal cells and premature cell death, which confirmed its role in cell division, differentiation and death (Lee et al. 2013). *E3 ubiquitin*-*protein ligase SINAT5*-*like* regulates auxin signals in plant cells thereby controlling plant development (Xie et al. 2002). The *TIMELESS*-*interacting protein* was shown to be involved in both the circadian rhythm and cell cycle checkpoint regulation (Yoshizawa-Sugata and Masai, 2007; Engelen et al. 2013). Knockdown of *TIMELESS*-*interacting protein* or the presence of mutations in the gene that lead to modification in the protein were associated with inefficient progression of S phase and DNA synthesis during the cell cycle (Yoshizawa-Sugata and Masai 2007). We can therefore hypothesize that the presence of missense mutations in the *TIMELESS*-*interacting protein* and *MYB*-*related protein 308*-*like* genes that are involved in cell cycle regulation possibly contribute to the variation in fruit filling in banana.

Our results suggest that continued cell division and expansion in the female inflorescence are key processes in determining the sink capacity of banana fruits. The actual mechanisms and gene pathways involved in the control of bunch weight and its component traits remain to be investigated. We can only hypothesize that genotypes with low, or abolished expression of genes involved in floral cell division, differentiation and expansion are likely to have a smaller number of cells in fruits to store the photosynthetic products, which results in poor fruit filling and low bunch weight. In crops such as wheat and rice, grain yield was reported to be controlled by grain weight gene *OsGW* (rice) and *TaGW2* (wheat), which determine seed size by modulating cell number and length (Zhang et al. 2018). In tomato, fruit weight was correlated with the number of cells due to cell division, cell expansion and endopolyploidization (Cheniclet et al. 2005).

The clustering of transcription factors and cell cycle regulation genes on chromosome 3 region that had SNPs associated with bunch weight and its component trait supported our hypothesis that fruit filling in this population was under control of a few QTL with major effects. This could also explain the high broad sense heritability of these traits, which were above 95%. However, given the size of our population, the resolution could have been limited. A future research should use a larger population to capture more recombination events on chromosome 3 so as to underpin the causal loci and unveil other loci that could have been missed out. Further research should also target populations with different genetic background such as Mchare, or plantain to verify if the loci controlling bunch weight and its components traits are conserved across the groups of *Musa* spp.. Martin et al. (2017) showed that heterozygous reciprocal translocations between chromosomes 1 and 4 resulted in segregation distortion, reduced recombination and linkage in the distal segments of the two chromosomes in the progeny of *Musa acuminata* ssp. *malaccensis*. A recent research by Dupouy et al. (2019) also identified two large reciprocal translocations in *Musa acuminata* ssp. *burmanica* involving chromosomes 2 and 8, and chromosomes 1 and 9. Studies comparing structural rearrangements and selection sweeps in the banana genome are required to shed more light on possible selection pressure that some regions underwent during domestication and their effects on traits such as yield. This could help to explain why most of the SNPs significantly associated with bunch weight and its component traits were clustered on chromosome 3. We expect that these results will stimulate further research in banana-breeding populations and diversity panels to underpin causal mutations that underlie the variations in bunch weight and its components in addition to other traits. More importantly, research geared towards the development of molecular markers in regions with significant SNPs could facilitate marker-assisted breeding and increase breeding speed to achieve high genetic gains.

## Electronic supplementary material

Below is the link to the electronic supplementary material.

Supplementary material 1 (HTML 357 kb)

Supplementary material 2 (PDF 455 kb)

Supplementary material 3 (TIFF 288 kb)

Supplementary material 4 (TIFF 435 kb)

Supplementary material 5 (TIFF 277 kb)

Supplementary material 6 (TIFF 211 kb)

Supplementary material 7 (XLSX 14 kb)

Supplementary material 8 (XLSX 59 kb)

Supplementary material 9 (XLSX 20 kb)

Supplementary material 10 (XLSX 16 kb)

Supplementary material 11 (XLSX 13 kb)

Supplementary material 12 (XLSX 13 kb)
