# Supplementary material for: Association genetics of bunch weight and its component traits in East African highland banana (Musa spp. AAA group)
Source: Theor Appl Genet. 2019 Sep 16;132(12):3295–308. doi: 10.1007/s00122-019-03425-x (PMC6820618; doi:10.1007/s00122-019-03425-x)
Supplement: Supplementary file 1 — Supplementary material 1 (HTML 357 kb) [file 122_2019_3425_MOESM1_ESM.html]

### SnpEff: Variant analysis

|  |
| --- |
| **Contents** Summary   Variant rate by chromosome  Variants by type   Number of variants by impact    Number of variants by functional class    Number of variants by effect   Quality histogram  InDel length histogram  Base variant table  Transition vs transversions (ts/tv)   Allele frequency    Allele Count    Codon change table    Amino acid change table    Chromosome variants plots    Details by gene |


---


**Summary**

|  |  |
| --- | --- |
| **Genome** | musa\_acuminata\_v2 |
| **Date** | 2019-06-29 17:42 |
| **SnpEff version** | ``` SnpEff 4.3t (build 2017-11-24 10:18), by Pablo Cingolani ``` |
| **Command line arguments** | ``` SnpEff  musa_acuminata_v2 /homes/nyine/GWAS/AllMerged_NoB_GWAS_for_snpeff.vcf ``` |
| **Warnings** | 12,832 |
| **Errors** | 0 |
| **Number of lines (input file)** | 27,178 |
| **Number of variants (before filter)** | 27,178 |
| **Number of not variants  (i.e. reference equals alternative)** | 0 |
| **Number of variants processed   (i.e. after filter and non-variants)** | 27,178 |
| **Number of known variants  (i.e. non-empty ID)** | 27,178 ( 100% ) |
| **Number of multi-allelic VCF entries  (i.e. more than two alleles)** | 0 |
| **Number of effects** | 84,057 |
| **Genome total length** | 450,848,473 |
| **Genome effective length** | 443,630,233 |
| **Variant rate** | 1 variant every 16,323 bases |


---


 **Variants rate details** 

| Chromosome | Length | Variants | Variants rate |
| --- | --- | --- | --- |
| 1 | 29,070,452 | 2,001 | 14,527 |
| 2 | 29,511,734 | 1,638 | 18,016 |
| 3 | 35,020,413 | 2,608 | 13,428 |
| 4 | 37,105,743 | 2,946 | 12,595 |
| 5 | 41,853,232 | 2,362 | 17,719 |
| 6 | 37,593,364 | 3,230 | 11,638 |
| 7 | 35,028,021 | 2,443 | 14,338 |
| 8 | 44,889,171 | 2,567 | 17,487 |
| 9 | 41,306,725 | 2,430 | 16,998 |
| 10 | 37,674,811 | 2,396 | 15,724 |
| 11 | 27,954,350 | 2,101 | 13,305 |
| Un\_random | 46,622,217 | 456 | 102,241 |
| Total | 443,630,233 | 27,178 | 16,323 |


---


 **Number variants by type**

| **Type** | **Total** |
| --- | --- |
| **SNP** | 27,178 |
| **MNP** | 0 |
| **INS** | 0 |
| **DEL** | 0 |
| **MIXED** | 0 |
| **INV** | 0 |
| **DUP** | 0 |
| **BND** | 0 |
| **INTERVAL** | 0 |
| **Total** | 27,178 |
| --- | --- |


---


 **Number of effects by impact** 

| **Type (alphabetical order)** |  | Count | Percent |
| --- | --- | --- | --- |
| **HIGH** |  | 209 | 0.249% |
| **LOW** |  | 6,434 | 7.654% |
| **MODERATE** |  | 6,437 | 7.658% |
| **MODIFIER** |  | 70,977 | 84.439% |


---


 **Number of effects by functional class** 

| **Type (alphabetical order)** |  | Count | Percent |
| --- | --- | --- | --- |
| **MISSENSE** |  | 6,488 | 53.373% |
| **NONSENSE** |  | 106 | 0.872% |
| **SILENT** |  | 5,562 | 45.755% |

  

Missense / Silent ratio: 1.1665


---


 **Number of effects by type and region** 

| Type | Region |
| --- | --- |
| | **Type (alphabetical order)** |  | Count | Percent | | --- | --- | --- | --- | | **3\_prime\_UTR\_variant** |  | 1,956 | 2.307% | | **5\_prime\_UTR\_premature\_start\_codon\_gain\_variant** |  | 122 | 0.144% | | **5\_prime\_UTR\_variant** |  | 861 | 1.015% | | **downstream\_gene\_variant** |  | 25,954 | 30.605% | | **intergenic\_region** |  | 8,195 | 9.664% | | **intron\_variant** |  | 12,354 | 14.568% | | **missense\_variant** |  | 6,437 | 7.591% | | **splice\_acceptor\_variant** |  | 34 | 0.04% | | **splice\_donor\_variant** |  | 18 | 0.021% | | **splice\_region\_variant** |  | 717 | 0.845% | | **stop\_gained** |  | 106 | 0.125% | | **stop\_lost** |  | 51 | 0.06% | | **stop\_retained\_variant** |  | 17 | 0.02% | | **synonymous\_variant** |  | 5,760 | 6.792% | | **upstream\_gene\_variant** |  | 22,220 | 26.202% | | | **Type (alphabetical order)** |  | Count | Percent | | --- | --- | --- | --- | | **DOWNSTREAM** |  | 25,954 | 30.877% | | **EXON** |  | 12,297 | 14.629% | | **INTERGENIC** |  | 8,195 | 9.749% | | **INTRON** |  | 11,791 | 14.027% | | **SPLICE\_SITE\_ACCEPTOR** |  | 34 | 0.04% | | **SPLICE\_SITE\_DONOR** |  | 18 | 0.021% | | **SPLICE\_SITE\_REGION** |  | 609 | 0.725% | | **UPSTREAM** |  | 22,220 | 26.434% | | **UTR\_3\_PRIME** |  | 1,956 | 2.327% | | **UTR\_5\_PRIME** |  | 983 | 1.169% | |


---


 **Quality:**

```
	
```


---


 **Insertions and deletions length:**

```
	
```


---


 **Base changes (SNPs)** 

|  |  |  |  |  |
| --- | --- | --- | --- | --- |
|  | **A** | **C** | **G** | **T** |
| **A** | 0 | 1,187 | 3,444 | 1,723 |
| **C** | 1,633 | 0 | 1,359 | 4,364 |
| **G** | 4,287 | 1,304 | 0 | 1,670 |
| **T** | 1,619 | 3,381 | 1,207 | 0 |

---


  **Ts/Tv (transitions / transversions)** 

**Note:** Only SNPs are used for this statistic.  
**Note:** This Ts/Tv ratio is a 'raw' ratio (ratio of observed events).

|  |  |
| --- | --- |
| Transitions | 2,937,701 |
| Transversions | 2,144,917 |
| Ts/Tv ratio | 1.3696 |

**All variants:**

```
Sample ,1201K_1,12419S_13,12468S_18,12468S_6,12477S_13,12479S_1,12479S_13,12618S_1,12949S_2,13284S_1,13522S_5,13573S_1,1438K_1,14539S_4,16191S_6,16242S_1,16285S_13,16285S_3,16285S_6,16285S_8,16457S_2,17503S_3,1968_2,19798S_2,21086S_1,2180K_6,222K_1,22598S_2,24410S_2,24434S_3,24583S_2,24797S_7,24928S_21,24928S_22,24948S_10,24948S_12,24948S_13,24948S_2,24948S_24,24948S_27,24948S_29,24948S_9,25031S_1,25031S_15,25031S_16,25031S_17,25031S_19,25031S_27,25031S_33,25031S_34,25031S_7,25066S_1,25066S_2,25089S_4,25102S_1,25117S_1,25117S_2,25117S_3,25328S_3,25356S_1,25371S_2,25435S_11,25435S_4,25457S_1,25474S_1,25474S_5,25499S_7,25508S_1,25583S_2,25623S_11,25628S_11,25653S_3,25680S_11,25680S_13,25737S_1,25909S_3,25974S_11,25974S_13,25974S_15,25974S_17,25974S_18,25974S_19,25974S_21,25974S_30,25974S_31,25974S_35,25974S_X,26039S_2,26060S_1,26075S_6,26075S_7,26075S_8,26224S_2,26224S_3,26260S_3,26288S_4,26315S_1,26315S_3,26316S_14,26316S_7,26317S_1,26337S_11A,26337S_11B,26337S_2,26337S_22A,26337S_22B,26337S_28,26337S_32,26337S_34,26337S_37,26337S_39,26337S_40,26337S_43,26363S_1,26369S_4,26369S_8,26466S_2,26466S_5,26530S_1,26540S_182,26660S_1,26666S_1,26709S_1,26725S_1,26815S_3,26815S_8,26815S_9,26820S_1,26840S_10,26840S_5,26840S_7,26840S_9,26874S_5,26975S_1,26975S_2,26990S_10,26990S_11,26990S_4,26998S_1,27073S_1,27074S_1,27184S_4,27184S_6,27184S_8,27261S_1,27261S_10,27261S_11,27262S_1,27262S_3,27264S_2,27334S_5,27346S_2,27346S_4,27401S_1,27437S_1,27494S_12,27494S_4,27494S_5,27524S_12A,27524S_12B,27524S_22,27524S_30,27528S_1,27579S_1,27579S_3,27684S_5,27713S_1,27744S_1,27770S_20,27770S_4,27825S_4,27833S_10,27833S_13,278732_12,27873S_14,27873S_17,27873S_18,27873S_26,27873S_3,27873S_33,27873S_37,27873S_38,27873S_4,27873S_5,27873S_7,27885S_1,27885S_9,27886S_5,27914S_1,27914S_11,27914S_13,27914S_18,27914S_21,27914S_22,27914S_24,27914S_26,27914S_3,27914S_6,27914S_7,27914S_8,279155_2,27915S_3,27935S_7,27960S_1,28030S_2,28030S_6,28033S_14,28033S_15,28033S_18,28033S_23,28033S_3,28033S_9,28036S_11,28036S_2,28060S_8,28068S_9,28071S_1,28073S_1,28077S_5,28095S_1,28164S_15,28164S_3,28165S_1,28188S_2,28200S_3,28246S_4,28246S_7,28256S_1,28257S_1,28257S_2,28257S_4,28260S_2,28263S_2,28432S_19,28432S_20,28432S_3,28434S_9,28452S_11,28465S_2,28465S_21,28476S_7,28476S_8,28479S_2,28481S_1,28492S_1,28506S_1,28528S_1,28561S_2,28561S_5,28780S_1,28974S_11,28974S_15,28974S_22,28974S_29,29114S_14A,29114S_14B,29114S_19,29114S_24,29165S_5,29275S_1,29285S_20,29364S_2,29586S_4,29636S_1,29725S_4,29725S_5,365K_1,376K_7,401K_1,5610S_1,660K_1,7197_2,7798S_2,8075_7,8386S_19,861S_1,9128_3,917K_2,9187S_8,9494S_10,9750S_13,C_V_rose,Calcutta_4,Entukura,Enyeru,Enzirabahima,HB,HJ,HX,Kabucuragye,Kazirakwe,Kokopo,Long_Tavoy,Malaccensis,Nakasabira,Nakawere,Nakayonga,Namwezi,Nante,Nfuuka,P_lilin,SH3142,SH3217,SH3362,Tereza,Total
Transitions ,10468,9559,9570,9864,9832,10616,10542,10123,10132,10248,9577,9578,10642,9909,10119,9629,10388,10302,10219,10237,10481,10044,9862,10045,9418,10206,10853,9570,9168,9782,9781,10248,10503,9990,9366,10375,9619,10435,10260,10854,9408,10209,9934,9391,9887,10302,10347,10309,9784,9867,10131,9922,9693,9775,10191,9654,10203,9942,9918,10257,9744,9639,9529,10365,10156,10785,9835,9372,10021,10315,10341,9865,9495,10214,9963,10335,9006,9716,9311,9643,8910,10242,10045,9579,9680,9526,9541,10719,9624,9928,10238,9928,9185,9660,10531,9104,10050,9836,10122,8861,10089,9339,9691,9176,9536,9746,10030,10106,9262,9094,9775,9449,9706,8752,10397,10242,7445,10228,9618,9347,10151,9129,10963,9927,10543,10240,9410,10502,9382,9937,9656,9828,9448,9297,9300,10108,9765,7588,8751,9368,8611,9141,9107,9498,9811,9389,9480,9950,9891,9220,9570,9173,9190,8792,9450,9527,9032,10142,9152,8863,9062,9195,9065,8677,8731,10078,9146,9228,9791,9706,9103,9414,9220,8988,9049,9153,9235,9242,8949,9071,8941,8947,9649,9489,9071,9277,9128,9171,9409,9140,9303,9279,9708,9645,9519,9366,9860,9636,8996,9461,8301,9763,9044,9179,8985,8935,9228,9015,9003,9463,9176,9046,9766,9338,9075,9328,9032,9603,9714,9465,10145,9392,9915,9565,8794,9245,9836,9430,9083,8958,9417,10116,9930,9381,9370,9132,9709,8962,9136,9028,10046,9774,9103,9292,8786,8930,10032,9089,9266,9423,9231,9192,9364,9280,8849,7776,8644,8672,9129,10724,9840,9368,10359,10632,10719,11157,10030,10814,10669,9837,10536,8848,9414,9742,9753,9996,9242,10889,9471,9792,9432,7515,10484,10808,10563,10419,10109,10050,10096,10682,10212,9348,9231,6472,10538,10334,10799,10871,10472,10472,7186,8800,8074,8467,10649,2937701
Transversions ,7671,7120,6973,7102,7142,7657,7630,7528,7349,7508,6975,7011,7878,7339,7529,7095,7606,7608,7503,7496,7752,7426,7278,7312,6917,7479,7806,6968,6703,7106,6989,7563,7583,7331,6983,7548,7065,7634,7581,7697,6766,7286,7003,7016,7181,7508,7535,7442,7203,7187,7359,7102,6951,7223,7392,7169,7478,7180,7448,7405,7067,6991,6993,7483,7343,7795,7253,7109,7217,7546,7464,7231,6943,7313,7251,7475,6608,7135,6862,7135,6597,7464,7276,7109,7151,7020,7024,7671,7035,7335,7286,7158,6829,7301,7604,6658,7439,7153,7299,6726,7318,6918,7248,6836,7043,7013,7344,7288,6944,6714,7181,6943,6959,6464,7494,7553,5429,7612,7070,6730,7461,6837,8000,7322,7613,7348,6971,7635,6744,7347,7084,7131,6936,6810,6791,7296,7238,5646,6383,6745,6421,6844,6607,6992,7102,6793,6834,7316,7157,6694,6995,6883,6736,6656,6933,6803,6574,7350,6735,6442,6631,6719,6605,6384,6485,7359,6814,6708,6988,7051,6624,7025,6665,6565,6786,6770,6577,6581,6522,6823,6574,6612,6865,6699,6537,6826,6642,6665,6828,6771,6890,6750,6991,7022,6871,6781,7094,7097,6574,6863,6233,7006,6737,6659,6555,6485,6772,6573,6736,6825,6639,6795,7099,6592,6602,7018,6682,6949,6919,6933,7212,6979,7201,6835,6455,6748,7130,6738,6576,6566,6679,7458,7310,6842,6766,6649,6892,6610,6611,6566,7200,6995,6654,6696,6403,6643,7142,6566,6740,6665,6662,6655,6686,6702,6413,5765,6405,6327,6627,7797,7113,6829,7709,7751,7654,8059,7396,7784,7813,7336,7621,6508,6848,7140,7171,7166,6674,7826,6993,7195,6934,5591,7741,7862,7680,7527,7309,7374,7349,7765,7660,6754,6910,4763,7769,7563,7957,7838,7780,7660,5125,6429,6045,6334,7673,2144917
Ts/Tv ,1.365,1.343,1.372,1.389,1.377,1.386,1.382,1.345,1.379,1.365,1.373,1.366,1.351,1.350,1.344,1.357,1.366,1.354,1.362,1.366,1.352,1.353,1.355,1.374,1.362,1.365,1.390,1.373,1.368,1.377,1.399,1.355,1.385,1.363,1.341,1.375,1.362,1.367,1.353,1.410,1.390,1.401,1.419,1.339,1.377,1.372,1.373,1.385,1.358,1.373,1.377,1.397,1.394,1.353,1.379,1.347,1.364,1.385,1.332,1.385,1.379,1.379,1.363,1.385,1.383,1.384,1.356,1.318,1.389,1.367,1.385,1.364,1.368,1.397,1.374,1.383,1.363,1.362,1.357,1.352,1.351,1.372,1.381,1.347,1.354,1.357,1.358,1.397,1.368,1.354,1.405,1.387,1.345,1.323,1.385,1.367,1.351,1.375,1.387,1.317,1.379,1.350,1.337,1.342,1.354,1.390,1.366,1.387,1.334,1.354,1.361,1.361,1.395,1.354,1.387,1.356,1.371,1.344,1.360,1.389,1.361,1.335,1.370,1.356,1.385,1.394,1.350,1.376,1.391,1.353,1.363,1.378,1.362,1.365,1.369,1.385,1.349,1.344,1.371,1.389,1.341,1.336,1.378,1.358,1.381,1.382,1.387,1.360,1.382,1.377,1.368,1.333,1.364,1.321,1.363,1.400,1.374,1.380,1.359,1.376,1.367,1.369,1.372,1.359,1.346,1.369,1.342,1.376,1.401,1.377,1.374,1.340,1.383,1.369,1.333,1.352,1.404,1.404,1.372,1.329,1.360,1.353,1.406,1.416,1.388,1.359,1.374,1.376,1.378,1.350,1.350,1.375,1.389,1.374,1.385,1.381,1.390,1.358,1.368,1.379,1.332,1.394,1.342,1.378,1.371,1.378,1.363,1.372,1.337,1.387,1.382,1.331,1.376,1.417,1.375,1.329,1.352,1.382,1.404,1.365,1.407,1.346,1.377,1.399,1.362,1.370,1.380,1.400,1.381,1.364,1.410,1.356,1.358,1.371,1.385,1.373,1.409,1.356,1.382,1.375,1.395,1.397,1.368,1.388,1.372,1.344,1.405,1.384,1.375,1.414,1.386,1.381,1.401,1.385,1.380,1.349,1.350,1.371,1.378,1.375,1.383,1.372,1.344,1.372,1.400,1.384,1.356,1.389,1.366,1.341,1.382,1.360,1.375,1.364,1.360,1.395,1.385,1.391,1.354,1.361,1.360,1.344,1.354,1.375,1.375,1.384,1.383,1.363,1.374,1.376,1.333,1.384,1.336,1.359,1.356,1.366,1.357,1.387,1.346,1.367,1.402,1.369,1.336,1.337,1.388,1.370
```

**Only known variants** (i.e. the ones having a non-empty ID field):

```
Sample ,1201K_1,12419S_13,12468S_18,12468S_6,12477S_13,12479S_1,12479S_13,12618S_1,12949S_2,13284S_1,13522S_5,13573S_1,1438K_1,14539S_4,16191S_6,16242S_1,16285S_13,16285S_3,16285S_6,16285S_8,16457S_2,17503S_3,1968_2,19798S_2,21086S_1,2180K_6,222K_1,22598S_2,24410S_2,24434S_3,24583S_2,24797S_7,24928S_21,24928S_22,24948S_10,24948S_12,24948S_13,24948S_2,24948S_24,24948S_27,24948S_29,24948S_9,25031S_1,25031S_15,25031S_16,25031S_17,25031S_19,25031S_27,25031S_33,25031S_34,25031S_7,25066S_1,25066S_2,25089S_4,25102S_1,25117S_1,25117S_2,25117S_3,25328S_3,25356S_1,25371S_2,25435S_11,25435S_4,25457S_1,25474S_1,25474S_5,25499S_7,25508S_1,25583S_2,25623S_11,25628S_11,25653S_3,25680S_11,25680S_13,25737S_1,25909S_3,25974S_11,25974S_13,25974S_15,25974S_17,25974S_18,25974S_19,25974S_21,25974S_30,25974S_31,25974S_35,25974S_X,26039S_2,26060S_1,26075S_6,26075S_7,26075S_8,26224S_2,26224S_3,26260S_3,26288S_4,26315S_1,26315S_3,26316S_14,26316S_7,26317S_1,26337S_11A,26337S_11B,26337S_2,26337S_22A,26337S_22B,26337S_28,26337S_32,26337S_34,26337S_37,26337S_39,26337S_40,26337S_43,26363S_1,26369S_4,26369S_8,26466S_2,26466S_5,26530S_1,26540S_182,26660S_1,26666S_1,26709S_1,26725S_1,26815S_3,26815S_8,26815S_9,26820S_1,26840S_10,26840S_5,26840S_7,26840S_9,26874S_5,26975S_1,26975S_2,26990S_10,26990S_11,26990S_4,26998S_1,27073S_1,27074S_1,27184S_4,27184S_6,27184S_8,27261S_1,27261S_10,27261S_11,27262S_1,27262S_3,27264S_2,27334S_5,27346S_2,27346S_4,27401S_1,27437S_1,27494S_12,27494S_4,27494S_5,27524S_12A,27524S_12B,27524S_22,27524S_30,27528S_1,27579S_1,27579S_3,27684S_5,27713S_1,27744S_1,27770S_20,27770S_4,27825S_4,27833S_10,27833S_13,278732_12,27873S_14,27873S_17,27873S_18,27873S_26,27873S_3,27873S_33,27873S_37,27873S_38,27873S_4,27873S_5,27873S_7,27885S_1,27885S_9,27886S_5,27914S_1,27914S_11,27914S_13,27914S_18,27914S_21,27914S_22,27914S_24,27914S_26,27914S_3,27914S_6,27914S_7,27914S_8,279155_2,27915S_3,27935S_7,27960S_1,28030S_2,28030S_6,28033S_14,28033S_15,28033S_18,28033S_23,28033S_3,28033S_9,28036S_11,28036S_2,28060S_8,28068S_9,28071S_1,28073S_1,28077S_5,28095S_1,28164S_15,28164S_3,28165S_1,28188S_2,28200S_3,28246S_4,28246S_7,28256S_1,28257S_1,28257S_2,28257S_4,28260S_2,28263S_2,28432S_19,28432S_20,28432S_3,28434S_9,28452S_11,28465S_2,28465S_21,28476S_7,28476S_8,28479S_2,28481S_1,28492S_1,28506S_1,28528S_1,28561S_2,28561S_5,28780S_1,28974S_11,28974S_15,28974S_22,28974S_29,29114S_14A,29114S_14B,29114S_19,29114S_24,29165S_5,29275S_1,29285S_20,29364S_2,29586S_4,29636S_1,29725S_4,29725S_5,365K_1,376K_7,401K_1,5610S_1,660K_1,7197_2,7798S_2,8075_7,8386S_19,861S_1,9128_3,917K_2,9187S_8,9494S_10,9750S_13,C_V_rose,Calcutta_4,Entukura,Enyeru,Enzirabahima,HB,HJ,HX,Kabucuragye,Kazirakwe,Kokopo,Long_Tavoy,Malaccensis,Nakasabira,Nakawere,Nakayonga,Namwezi,Nante,Nfuuka,P_lilin,SH3142,SH3217,SH3362,Tereza,Total
Transitions ,10468,9559,9570,9864,9832,10616,10542,10123,10132,10248,9577,9578,10642,9909,10119,9629,10388,10302,10219,10237,10481,10044,9862,10045,9418,10206,10853,9570,9168,9782,9781,10248,10503,9990,9366,10375,9619,10435,10260,10854,9408,10209,9934,9391,9887,10302,10347,10309,9784,9867,10131,9922,9693,9775,10191,9654,10203,9942,9918,10257,9744,9639,9529,10365,10156,10785,9835,9372,10021,10315,10341,9865,9495,10214,9963,10335,9006,9716,9311,9643,8910,10242,10045,9579,9680,9526,9541,10719,9624,9928,10238,9928,9185,9660,10531,9104,10050,9836,10122,8861,10089,9339,9691,9176,9536,9746,10030,10106,9262,9094,9775,9449,9706,8752,10397,10242,7445,10228,9618,9347,10151,9129,10963,9927,10543,10240,9410,10502,9382,9937,9656,9828,9448,9297,9300,10108,9765,7588,8751,9368,8611,9141,9107,9498,9811,9389,9480,9950,9891,9220,9570,9173,9190,8792,9450,9527,9032,10142,9152,8863,9062,9195,9065,8677,8731,10078,9146,9228,9791,9706,9103,9414,9220,8988,9049,9153,9235,9242,8949,9071,8941,8947,9649,9489,9071,9277,9128,9171,9409,9140,9303,9279,9708,9645,9519,9366,9860,9636,8996,9461,8301,9763,9044,9179,8985,8935,9228,9015,9003,9463,9176,9046,9766,9338,9075,9328,9032,9603,9714,9465,10145,9392,9915,9565,8794,9245,9836,9430,9083,8958,9417,10116,9930,9381,9370,9132,9709,8962,9136,9028,10046,9774,9103,9292,8786,8930,10032,9089,9266,9423,9231,9192,9364,9280,8849,7776,8644,8672,9129,10724,9840,9368,10359,10632,10719,11157,10030,10814,10669,9837,10536,8848,9414,9742,9753,9996,9242,10889,9471,9792,9432,7515,10484,10808,10563,10419,10109,10050,10096,10682,10212,9348,9231,6472,10538,10334,10799,10871,10472,10472,7186,8800,8074,8467,10649,2937701
Transversions ,7671,7120,6973,7102,7142,7657,7630,7528,7349,7508,6975,7011,7878,7339,7529,7095,7606,7608,7503,7496,7752,7426,7278,7312,6917,7479,7806,6968,6703,7106,6989,7563,7583,7331,6983,7548,7065,7634,7581,7697,6766,7286,7003,7016,7181,7508,7535,7442,7203,7187,7359,7102,6951,7223,7392,7169,7478,7180,7448,7405,7067,6991,6993,7483,7343,7795,7253,7109,7217,7546,7464,7231,6943,7313,7251,7475,6608,7135,6862,7135,6597,7464,7276,7109,7151,7020,7024,7671,7035,7335,7286,7158,6829,7301,7604,6658,7439,7153,7299,6726,7318,6918,7248,6836,7043,7013,7344,7288,6944,6714,7181,6943,6959,6464,7494,7553,5429,7612,7070,6730,7461,6837,8000,7322,7613,7348,6971,7635,6744,7347,7084,7131,6936,6810,6791,7296,7238,5646,6383,6745,6421,6844,6607,6992,7102,6793,6834,7316,7157,6694,6995,6883,6736,6656,6933,6803,6574,7350,6735,6442,6631,6719,6605,6384,6485,7359,6814,6708,6988,7051,6624,7025,6665,6565,6786,6770,6577,6581,6522,6823,6574,6612,6865,6699,6537,6826,6642,6665,6828,6771,6890,6750,6991,7022,6871,6781,7094,7097,6574,6863,6233,7006,6737,6659,6555,6485,6772,6573,6736,6825,6639,6795,7099,6592,6602,7018,6682,6949,6919,6933,7212,6979,7201,6835,6455,6748,7130,6738,6576,6566,6679,7458,7310,6842,6766,6649,6892,6610,6611,6566,7200,6995,6654,6696,6403,6643,7142,6566,6740,6665,6662,6655,6686,6702,6413,5765,6405,6327,6627,7797,7113,6829,7709,7751,7654,8059,7396,7784,7813,7336,7621,6508,6848,7140,7171,7166,6674,7826,6993,7195,6934,5591,7741,7862,7680,7527,7309,7374,7349,7765,7660,6754,6910,4763,7769,7563,7957,7838,7780,7660,5125,6429,6045,6334,7673,2144917
Ts/Tv ,1.365,1.343,1.372,1.389,1.377,1.386,1.382,1.345,1.379,1.365,1.373,1.366,1.351,1.350,1.344,1.357,1.366,1.354,1.362,1.366,1.352,1.353,1.355,1.374,1.362,1.365,1.390,1.373,1.368,1.377,1.399,1.355,1.385,1.363,1.341,1.375,1.362,1.367,1.353,1.410,1.390,1.401,1.419,1.339,1.377,1.372,1.373,1.385,1.358,1.373,1.377,1.397,1.394,1.353,1.379,1.347,1.364,1.385,1.332,1.385,1.379,1.379,1.363,1.385,1.383,1.384,1.356,1.318,1.389,1.367,1.385,1.364,1.368,1.397,1.374,1.383,1.363,1.362,1.357,1.352,1.351,1.372,1.381,1.347,1.354,1.357,1.358,1.397,1.368,1.354,1.405,1.387,1.345,1.323,1.385,1.367,1.351,1.375,1.387,1.317,1.379,1.350,1.337,1.342,1.354,1.390,1.366,1.387,1.334,1.354,1.361,1.361,1.395,1.354,1.387,1.356,1.371,1.344,1.360,1.389,1.361,1.335,1.370,1.356,1.385,1.394,1.350,1.376,1.391,1.353,1.363,1.378,1.362,1.365,1.369,1.385,1.349,1.344,1.371,1.389,1.341,1.336,1.378,1.358,1.381,1.382,1.387,1.360,1.382,1.377,1.368,1.333,1.364,1.321,1.363,1.400,1.374,1.380,1.359,1.376,1.367,1.369,1.372,1.359,1.346,1.369,1.342,1.376,1.401,1.377,1.374,1.340,1.383,1.369,1.333,1.352,1.404,1.404,1.372,1.329,1.360,1.353,1.406,1.416,1.388,1.359,1.374,1.376,1.378,1.350,1.350,1.375,1.389,1.374,1.385,1.381,1.390,1.358,1.368,1.379,1.332,1.394,1.342,1.378,1.371,1.378,1.363,1.372,1.337,1.387,1.382,1.331,1.376,1.417,1.375,1.329,1.352,1.382,1.404,1.365,1.407,1.346,1.377,1.399,1.362,1.370,1.380,1.400,1.381,1.364,1.410,1.356,1.358,1.371,1.385,1.373,1.409,1.356,1.382,1.375,1.395,1.397,1.368,1.388,1.372,1.344,1.405,1.384,1.375,1.414,1.386,1.381,1.401,1.385,1.380,1.349,1.350,1.371,1.378,1.375,1.383,1.372,1.344,1.372,1.400,1.384,1.356,1.389,1.366,1.341,1.382,1.360,1.375,1.364,1.360,1.395,1.385,1.391,1.354,1.361,1.360,1.344,1.354,1.375,1.375,1.384,1.383,1.363,1.374,1.376,1.333,1.384,1.336,1.359,1.356,1.366,1.357,1.387,1.346,1.367,1.402,1.369,1.336,1.337,1.388,1.370
```

---


  **Allele frequency** 
  

|  |  |
| --- | --- |
| Min | 1 |
| Max | 98 |
| Mean | 30.164 |
| Median | 18 |
| Standard deviation | 27.928 |
| Values | 1,2,3,4,5,6,7,8,9,10,11,12,13,14,15,16,17,18,19,20,21,22,23,24,25,26,27,28,29,30,31,32,33,34,35,36,37,38,39,40,41,42,43,44,45,46,47,48,49,50,51,52,53,54,55,56,57,58,59,60,61,62,63,64,65,66,67,68,69,70,71,72,73,74,75,76,77,78,79,80,81,82,83,84,85,86,87,88,89,90,91,92,93,94,95,96,97,98 |
| Count | 97,402,708,899,1454,1129,1016,962,940,1004,761,793,667,654,599,558,507,502,426,529,432,390,380,364,306,276,273,289,224,283,229,216,209,230,177,176,185,212,160,186,153,157,132,149,124,138,111,102,91,105,113,109,108,97,94,92,92,100,89,112,107,100,103,104,97,108,98,104,97,152,116,104,123,123,102,116,118,140,156,197,115,148,146,148,130,149,146,167,201,229,202,201,210,196,158,123,64,8 |

---


  **Allele Count** 
  

|  |  |
| --- | --- |
| Min | 8 |
| Max | 599 |
| Mean | 187.012 |
| Median | 115 |
| Standard deviation | 170.347 |
| Values | 8,9,10,11,12,13,14,15,16,17,18,19,20,21,22,23,24,25,26,27,28,29,30,31,32,33,34,35,36,37,38,39,40,41,42,43,44,45,46,47,48,49,50,51,52,53,54,55,56,57,58,59,60,61,62,63,64,65,66,67,68,69,70,71,72,73,74,75,76,77,78,79,80,81,82,83,84,85,86,87,88,89,90,91,92,93,94,95,96,97,98,99,100,101,102,103,104,105,106,107,108,109,110,111,112,113,114,115,116,117,118,119,120,121,122,123,124,125,126,127,128,129,130,131,132,133,134,135,136,137,138,139,140,141,142,143,144,145,146,147,148,149,150,151,152,153,154,155,156,157,158,159,160,161,162,163,164,165,166,167,168,169,170,171,172,173,174,175,176,177,178,179,180,181,182,183,184,185,186,187,188,189,190,191,192,193,194,195,196,197,198,199,200,201,202,203,204,205,206,207,208,209,210,211,212,213,214,215,216,217,218,219,220,221,222,223,224,225,226,227,228,229,230,231,232,233,234,235,236,237,238,239,240,241,242,243,244,245,246,247,248,249,250,251,252,253,254,255,256,257,258,259,260,261,262,263,264,265,266,267,268,269,270,271,272,273,274,275,276,277,278,279,280,281,282,283,284,285,286,287,288,289,290,291,292,293,294,295,296,297,298,299,300,301,302,303,304,305,306,307,308,309,310,311,312,313,314,315,316,317,318,319,320,321,322,323,324,325,326,327,328,329,330,331,332,333,334,335,336,337,338,339,340,341,342,343,344,345,346,347,348,349,350,351,352,353,354,355,356,357,358,359,360,361,362,363,364,365,366,367,368,369,370,371,372,373,374,375,376,377,378,379,380,381,382,383,384,385,386,387,388,389,390,391,392,393,394,395,396,397,398,399,400,401,402,403,404,405,406,407,408,409,410,411,412,413,414,415,416,417,418,419,420,421,422,423,424,425,426,427,428,429,430,431,432,433,434,435,436,437,438,439,440,441,442,443,444,445,446,447,448,449,450,451,452,453,454,455,456,457,458,459,460,461,462,463,464,465,466,467,468,469,470,471,472,473,474,475,476,477,478,479,480,481,482,483,484,485,486,487,488,489,490,491,492,493,494,495,496,497,498,499,500,501,502,503,504,505,506,507,508,509,510,511,512,513,514,515,516,517,518,519,520,521,522,523,524,525,526,527,528,529,530,531,532,533,534,535,536,537,538,539,540,541,542,543,544,545,546,547,548,549,550,551,552,553,554,555,556,557,558,559,560,561,562,563,564,565,566,567,568,569,570,571,572,573,574,575,576,577,578,579,580,581,582,583,584,585,586,587,588,589,590,591,592,593,594,595,596,597,598,599 |
| Count | 3,6,14,30,44,50,41,70,66,84,91,101,104,112,123,122,146,114,132,169,144,154,186,234,206,254,254,246,260,219,159,220,183,178,170,188,160,171,163,157,177,157,158,154,174,146,173,149,162,168,146,164,151,141,172,148,156,125,148,114,130,147,125,119,125,115,142,128,141,149,117,116,119,118,93,109,127,101,112,101,104,114,113,110,91,101,88,116,93,110,97,85,111,84,81,100,80,89,87,83,78,90,89,68,78,89,88,90,79,55,76,75,66,75,79,74,69,63,107,70,67,67,75,72,68,79,71,53,57,60,91,64,65,72,63,74,55,54,62,61,53,66,42,75,67,48,47,52,42,63,54,47,52,32,44,49,52,39,56,37,46,44,51,52,45,54,49,50,39,29,29,45,44,24,53,40,41,44,37,46,42,33,41,38,47,33,34,36,30,44,34,25,41,42,30,33,41,41,34,30,43,35,36,42,37,37,32,34,29,29,24,29,26,33,36,33,31,17,35,29,24,33,35,29,35,35,34,40,37,31,20,29,33,18,30,30,25,24,29,30,27,19,32,28,29,14,24,25,33,31,33,23,24,22,24,25,24,24,19,19,21,29,29,30,23,19,19,21,17,19,19,24,24,33,22,25,21,12,25,15,25,17,13,24,17,16,9,21,21,18,17,23,17,15,15,11,10,16,16,11,16,12,20,14,15,25,20,21,15,17,24,23,19,14,11,18,20,16,23,22,11,16,15,16,9,23,16,18,13,23,18,14,11,15,19,22,11,13,16,11,11,10,17,22,17,15,11,19,16,20,13,21,12,16,23,15,6,17,11,14,22,26,13,13,13,15,11,18,23,24,16,12,20,17,18,20,13,14,14,15,20,24,16,20,19,10,14,15,26,26,13,14,16,16,12,12,24,15,20,16,21,15,16,17,14,19,17,19,20,11,19,16,19,10,16,14,13,23,21,25,22,16,26,18,20,25,21,22,19,20,18,16,18,13,15,14,25,19,15,24,21,23,18,22,17,24,17,16,29,20,15,21,21,12,16,17,22,18,23,13,22,18,23,20,14,21,24,16,21,18,28,24,28,21,29,25,23,28,33,18,27,27,31,25,30,29,28,19,23,22,16,12,23,31,22,19,28,25,23,19,27,26,25,23,26,24,30,27,21,24,22,23,24,23,20,20,20,17,25,17,25,30,35,16,23,20,25,40,22,37,33,18,26,25,28,30,19,41,41,37,33,26,45,36,39,33,26,24,36,29,34,28,39,36,38,43,25,39,27,29,34,31,36,33,39,37,35,32,31,36,27,35,29,33,24,30,21,21,20,25,30,20,11,17,12,11,10,13,10,8,6,2 |

---


  **Hom/Het per sample** 
  
  
  

```
Sample_names , 1201K_1, 12419S_13, 12468S_18, 12468S_6, 12477S_13, 12479S_1, 12479S_13, 12618S_1, 12949S_2, 13284S_1, 13522S_5, 13573S_1, 1438K_1, 14539S_4, 16191S_6, 16242S_1, 16285S_13, 16285S_3, 16285S_6, 16285S_8, 16457S_2, 17503S_3, 1968_2, 19798S_2, 21086S_1, 2180K_6, 222K_1, 22598S_2, 24410S_2, 24434S_3, 24583S_2, 24797S_7, 24928S_21, 24928S_22, 24948S_10, 24948S_12, 24948S_13, 24948S_2, 24948S_24, 24948S_27, 24948S_29, 24948S_9, 25031S_1, 25031S_15, 25031S_16, 25031S_17, 25031S_19, 25031S_27, 25031S_33, 25031S_34, 25031S_7, 25066S_1, 25066S_2, 25089S_4, 25102S_1, 25117S_1, 25117S_2, 25117S_3, 25328S_3, 25356S_1, 25371S_2, 25435S_11, 25435S_4, 25457S_1, 25474S_1, 25474S_5, 25499S_7, 25508S_1, 25583S_2, 25623S_11, 25628S_11, 25653S_3, 25680S_11, 25680S_13, 25737S_1, 25909S_3, 25974S_11, 25974S_13, 25974S_15, 25974S_17, 25974S_18, 25974S_19, 25974S_21, 25974S_30, 25974S_31, 25974S_35, 25974S_X, 26039S_2, 26060S_1, 26075S_6, 26075S_7, 26075S_8, 26224S_2, 26224S_3, 26260S_3, 26288S_4, 26315S_1, 26315S_3, 26316S_14, 26316S_7, 26317S_1, 26337S_11A, 26337S_11B, 26337S_2, 26337S_22A, 26337S_22B, 26337S_28, 26337S_32, 26337S_34, 26337S_37, 26337S_39, 26337S_40, 26337S_43, 26363S_1, 26369S_4, 26369S_8, 26466S_2, 26466S_5, 26530S_1, 26540S_182, 26660S_1, 26666S_1, 26709S_1, 26725S_1, 26815S_3, 26815S_8, 26815S_9, 26820S_1, 26840S_10, 26840S_5, 26840S_7, 26840S_9, 26874S_5, 26975S_1, 26975S_2, 26990S_10, 26990S_11, 26990S_4, 26998S_1, 27073S_1, 27074S_1, 27184S_4, 27184S_6, 27184S_8, 27261S_1, 27261S_10, 27261S_11, 27262S_1, 27262S_3, 27264S_2, 27334S_5, 27346S_2, 27346S_4, 27401S_1, 27437S_1, 27494S_12, 27494S_4, 27494S_5, 27524S_12A, 27524S_12B, 27524S_22, 27524S_30, 27528S_1, 27579S_1, 27579S_3, 27684S_5, 27713S_1, 27744S_1, 27770S_20, 27770S_4, 27825S_4, 27833S_10, 27833S_13, 278732_12, 27873S_14, 27873S_17, 27873S_18, 27873S_26, 27873S_3, 27873S_33, 27873S_37, 27873S_38, 27873S_4, 27873S_5, 27873S_7, 27885S_1, 27885S_9, 27886S_5, 27914S_1, 27914S_11, 27914S_13, 27914S_18, 27914S_21, 27914S_22, 27914S_24, 27914S_26, 27914S_3, 27914S_6, 27914S_7, 27914S_8, 279155_2, 27915S_3, 27935S_7, 27960S_1, 28030S_2, 28030S_6, 28033S_14, 28033S_15, 28033S_18, 28033S_23, 28033S_3, 28033S_9, 28036S_11, 28036S_2, 28060S_8, 28068S_9, 28071S_1, 28073S_1, 28077S_5, 28095S_1, 28164S_15, 28164S_3, 28165S_1, 28188S_2, 28200S_3, 28246S_4, 28246S_7, 28256S_1, 28257S_1, 28257S_2, 28257S_4, 28260S_2, 28263S_2, 28432S_19, 28432S_20, 28432S_3, 28434S_9, 28452S_11, 28465S_2, 28465S_21, 28476S_7, 28476S_8, 28479S_2, 28481S_1, 28492S_1, 28506S_1, 28528S_1, 28561S_2, 28561S_5, 28780S_1, 28974S_11, 28974S_15, 28974S_22, 28974S_29, 29114S_14A, 29114S_14B, 29114S_19, 29114S_24, 29165S_5, 29275S_1, 29285S_20, 29364S_2, 29586S_4, 29636S_1, 29725S_4, 29725S_5, 365K_1, 376K_7, 401K_1, 5610S_1, 660K_1, 7197_2, 7798S_2, 8075_7, 8386S_19, 861S_1, 9128_3, 917K_2, 9187S_8, 9494S_10, 9750S_13, C_V_rose, Calcutta_4, Entukura, Enyeru, Enzirabahima, HB, HJ, HX, Kabucuragye, Kazirakwe, Kokopo, Long_Tavoy, Malaccensis, Nakasabira, Nakawere, Nakayonga, Namwezi, Nante, Nfuuka, P_lilin, SH3142, SH3217, SH3362, Tereza
Reference , 17239, 17166, 17964, 17069, 17902, 17215, 17436, 15488, 17714, 17578, 18006, 18043, 16597, 17282, 17998, 17941, 17760, 17647, 17361, 17970, 17294, 17255, 17735, 17657, 17552, 17423, 16214, 17986, 17884, 17841, 17706, 17471, 16833, 16143, 17184, 17053, 17164, 17164, 17590, 17180, 16601, 17658, 17984, 18128, 17859, 16719, 17673, 17756, 17029, 18086, 17772, 17903, 17967, 17584, 17190, 17781, 15389, 17781, 16419, 17417, 17812, 17947, 17380, 17536, 17691, 17243, 17834, 17980, 17577, 17540, 17519, 17973, 17639, 17757, 17885, 17505, 18468, 17224, 18235, 16941, 17222, 17583, 17758, 17350, 17629, 16796, 17174, 17255, 18012, 17684, 17451, 17778, 18008, 17828, 17202, 18000, 17176, 16653, 17827, 18452, 17799, 18039, 15725, 17719, 17947, 18114, 17684, 17621, 17968, 18334, 16360, 17079, 18114, 18689, 17278, 17551, 20101, 17225, 15867, 18563, 17230, 18315, 17051, 17326, 17295, 17635, 17518, 17344, 18254, 17730, 18306, 17938, 16789, 17894, 18253, 17261, 17875, 19758, 18737, 17598, 18706, 18358, 18468, 16573, 18057, 18157, 18138, 16473, 17861, 18562, 18132, 16700, 17670, 18175, 18216, 18291, 18291, 15443, 18361, 18555, 18490, 17775, 18514, 18362, 17991, 17513, 18444, 18422, 18128, 17736, 18497, 16471, 18464, 18237, 18105, 18296, 18465, 18603, 18668, 18373, 18443, 18435, 18162, 18104, 18348, 17592, 18497, 17727, 18239, 18399, 17504, 18009, 18112, 17611, 18140, 18227, 17238, 18117, 17320, 17133, 18596, 15643, 18345, 18478, 18517, 18515, 18285, 18581, 18037, 18275, 18474, 16305, 17755, 18390, 18547, 18217, 18265, 18015, 18115, 18137, 17694, 18110, 17757, 18231, 18352, 18403, 17993, 18206, 17726, 18028, 17995, 17527, 17307, 18116, 17522, 18441, 18132, 18444, 18213, 18415, 17901, 17213, 18529, 18378, 18767, 18482, 17871, 18581, 18368, 17423, 18414, 18326, 18431, 18419, 18538, 19849, 18490, 18855, 18523, 16569, 17782, 17813, 17120, 16961, 16440, 16677, 17726, 16521, 16236, 17502, 16889, 18820, 17976, 16154, 17479, 17662, 18501, 17009, 17804, 17718, 17628, 19473, 17738, 17281, 17481, 17021, 17782, 17744, 17741, 17126, 15863, 18756, 17340, 21120, 16985, 17353, 16510, 17174, 17057, 17507, 20584, 19129, 19551, 19058, 16973
Het , 1739, 3345, 1885, 3252, 1578, 1653, 1312, 5729, 1447, 1444, 1792, 1681, 2642, 2544, 712, 1750, 842, 1152, 1912, 683, 1535, 2376, 1746, 1685, 2917, 1825, 3269, 1846, 2717, 1786, 2174, 1603, 2604, 4749, 3639, 2327, 3344, 1959, 1335, 1445, 4980, 1545, 1451, 1693, 1570, 3108, 1128, 1093, 3311, 1130, 1322, 1526, 1778, 2190, 2393, 1971, 5897, 1672, 4152, 1860, 1921, 1832, 3074, 1436, 1475, 1290, 1600, 1915, 1964, 1415, 1513, 1314, 2640, 1315, 1372, 1536, 1806, 3057, 1713, 3696, 4405, 1484, 1519, 2968, 2267, 4218, 3443, 1456, 1673, 1725, 1930, 1714, 2326, 1739, 1817, 2594, 2515, 4061, 1281, 1865, 1351, 2021, 5967, 2906, 1883, 1369, 1614, 1720, 2214, 1880, 4680, 3806, 1463, 1762, 1909, 1459, 1280, 2066, 5934, 1153, 2284, 1760, 1291, 2455, 1610, 1498, 2939, 1531, 1722, 1612, 1004, 1521, 4394, 2461, 1759, 2430, 1603, 1606, 1748, 3047, 1912, 1655, 1706, 4720, 1329, 1860, 1766, 4144, 1586, 1318, 1527, 4900, 3090, 2558, 1541, 1444, 2168, 5978, 1747, 1941, 1683, 2892, 1658, 2571, 3158, 1893, 1508, 1576, 1321, 2127, 1635, 4975, 1543, 2329, 2311, 1841, 1614, 1327, 1549, 1716, 1955, 1927, 1518, 1960, 2052, 3069, 1592, 3066, 1641, 1647, 3155, 2309, 1433, 2467, 1686, 1755, 2926, 1389, 4146, 3766, 2630, 6301, 1885, 1562, 1782, 1906, 1786, 1606, 2543, 1518, 1593, 5905, 1981, 1646, 1585, 1576, 2112, 1774, 1493, 1684, 1611, 1765, 1726, 1494, 2403, 1557, 1404, 1776, 3245, 2776, 2270, 1728, 2502, 1901, 3176, 1693, 1491, 1896, 2183, 1932, 1308, 3161, 1541, 1612, 1633, 1819, 1440, 1539, 1614, 3422, 1635, 1857, 1444, 1536, 2018, 1117, 2327, 1647, 1554, 2697, 1839, 2533, 2048, 2051, 3103, 1786, 1478, 2716, 3402, 2179, 2421, 1360, 2142, 5166, 2474, 1870, 1438, 1623, 2284, 1933, 2734, 2304, 655, 1124, 1151, 2368, 1374, 1444, 1429, 1657, 4758, 742, 3535, 881, 2079, 1753, 2580, 1299, 1990, 1210, 877, 869, 1135, 1439, 2088
Hom , 8200, 6667, 7329, 6857, 7698, 8310, 8430, 5961, 8017, 8156, 7380, 7454, 7939, 7352, 8468, 7487, 8576, 8379, 7905, 8525, 8349, 7547, 7697, 7836, 6709, 7930, 7695, 7346, 6577, 7551, 7298, 8104, 7741, 6286, 6355, 7798, 6670, 8055, 8253, 8553, 5597, 7975, 7743, 7357, 7749, 7351, 8377, 8329, 6838, 7962, 8084, 7749, 7433, 7404, 7595, 7426, 5892, 7725, 6607, 7901, 7445, 7399, 6724, 8206, 8012, 8645, 7744, 7283, 7637, 8223, 8146, 7891, 6899, 8106, 7921, 8137, 6904, 6897, 7230, 6541, 5551, 8111, 7901, 6860, 7282, 6164, 6561, 8467, 7493, 7769, 7797, 7686, 6844, 7611, 8159, 6584, 7487, 6464, 8070, 6861, 8028, 7118, 5486, 6553, 7348, 7695, 7880, 7837, 6996, 6964, 6138, 6293, 7601, 6727, 7991, 8168, 5797, 7887, 5377, 7462, 7664, 7103, 8836, 7397, 8273, 8045, 6721, 8303, 7202, 7836, 7868, 7719, 5995, 6823, 7166, 7487, 7700, 5814, 6693, 6533, 6560, 7165, 7004, 5885, 7792, 7161, 7274, 6561, 7731, 7298, 7519, 5578, 6418, 6445, 7421, 7443, 6719, 5757, 7070, 6682, 7005, 6511, 7006, 6245, 6029, 7772, 7226, 7180, 7729, 7315, 7046, 5732, 7171, 6612, 6762, 7041, 7099, 7248, 6961, 7089, 6780, 6816, 7498, 7114, 6778, 6517, 7089, 6385, 7298, 7132, 6519, 6860, 7633, 7100, 7352, 7196, 7014, 7672, 5712, 6279, 5952, 5234, 6948, 7138, 6879, 6757, 7107, 6991, 6598, 7385, 7111, 4968, 7442, 7142, 7046, 7385, 6801, 7389, 7570, 7357, 7873, 7303, 7695, 7453, 6423, 7218, 7781, 7196, 6207, 6374, 6913, 7923, 7369, 7161, 6480, 7044, 7555, 6838, 6782, 6831, 7969, 6804, 7108, 7188, 6778, 6877, 7867, 7058, 7196, 6333, 7129, 6995, 7303, 7223, 6622, 6212, 6361, 6676, 7101, 7912, 7557, 6832, 8010, 8166, 7635, 8715, 7974, 7941, 7540, 7497, 7868, 6998, 7060, 5858, 7225, 7646, 7239, 8546, 7090, 7527, 6816, 5401, 8785, 8773, 8546, 7789, 8022, 7990, 8008, 8395, 6557, 7680, 6303, 5177, 8114, 8072, 8088, 8705, 8131, 8461, 5717, 7180, 6492, 6681, 8117
Missing , 0, 0, 0, 0, 0, 0, 0, 0, 0, 0, 0, 0, 0, 0, 0, 0, 0, 0, 0, 0, 0, 0, 0, 0, 0, 0, 0, 0, 0, 0, 0, 0, 0, 0, 0, 0, 0, 0, 0, 0, 0, 0, 0, 0, 0, 0, 0, 0, 0, 0, 0, 0, 0, 0, 0, 0, 0, 0, 0, 0, 0, 0, 0, 0, 0, 0, 0, 0, 0, 0, 0, 0, 0, 0, 0, 0, 0, 0, 0, 0, 0, 0, 0, 0, 0, 0, 0, 0, 0, 0, 0, 0, 0, 0, 0, 0, 0, 0, 0, 0, 0, 0, 0, 0, 0, 0, 0, 0, 0, 0, 0, 0, 0, 0, 0, 0, 0, 0, 0, 0, 0, 0, 0, 0, 0, 0, 0, 0, 0, 0, 0, 0, 0, 0, 0, 0, 0, 0, 0, 0, 0, 0, 0, 0, 0, 0, 0, 0, 0, 0, 0, 0, 0, 0, 0, 0, 0, 0, 0, 0, 0, 0, 0, 0, 0, 0, 0, 0, 0, 0, 0, 0, 0, 0, 0, 0, 0, 0, 0, 0, 0, 0, 0, 0, 0, 0, 0, 0, 0, 0, 0, 0, 0, 0, 0, 0, 0, 0, 0, 0, 0, 0, 0, 0, 0, 0, 0, 0, 0, 0, 0, 0, 0, 0, 0, 0, 0, 0, 0, 0, 0, 0, 0, 0, 0, 0, 0, 0, 0, 0, 0, 0, 0, 0, 0, 0, 0, 0, 0, 0, 0, 0, 0, 0, 0, 0, 0, 0, 0, 0, 0, 0, 0, 0, 0, 0, 0, 0, 0, 0, 0, 0, 0, 0, 0, 0, 0, 0, 0, 0, 0, 0, 0, 0, 0, 0, 0, 0, 0, 0, 0, 0, 0, 0, 0, 0, 0, 0, 0, 0, 0, 0, 0, 0, 0, 0, 0, 0, 0, 0, 0, 0, 0, 0, 0
```

---


 **Codon changes**

How to read this table:   
- Rows are reference codons and columns are changed codons. E.g. Row 'AAA' column 'TAA' indicates how many 'AAA' codons have been replaced by 'TAA' codons.  
- Red background colors indicate that more changes happened (heat-map).  
- Diagonals are indicated using grey background color   
- WARNING: This table may include different translation codon tables (e.g. mamalian DNA and mitochondrial DNA).

|  | AAA | AAC | AAG | AAT | ACA | ACC | ACG | ACT | AGA | AGC | AGG | AGT | ATA | ATC | ATG | ATT | CAA | CAC | CAG | CAT | CCA | CCC | CCG | CCT | CGA | CGC | CGG | CGT | CTA | CTC | CTG | CTT | GAA | GAC | GAG | GAT | GCA | GCC | GCG | GCT | GGA | GGC | GGG | GGT | GTA | GTC | GTG | GTT | TAA | TAC | TAG | TAT | TCA | TCC | TCG | TCT | TGA | TGC | TGG | TGT | TTA | TTC | TTG | TTT |
| --- | --- | --- | --- | --- | --- | --- | --- | --- | --- | --- | --- | --- | --- | --- | --- | --- | --- | --- | --- | --- | --- | --- | --- | --- | --- | --- | --- | --- | --- | --- | --- | --- | --- | --- | --- | --- | --- | --- | --- | --- | --- | --- | --- | --- | --- | --- | --- | --- | --- | --- | --- | --- | --- | --- | --- | --- | --- | --- | --- | --- | --- | --- | --- | --- |
| AAA | 3 | 20 | 47 | 10 | 13 |  |  |  | 22 |  |  |  | 18 |  |  |  | 17 |  |  |  |  |  |  |  |  |  |  |  |  |  |  |  | 27 |  |  |  |  |  |  |  |  |  |  |  |  |  |  |  | 4 |  |  |  |  |  |  |  |  |  |  |  |  |  |  |  |
| AAC | 9 | 1 | 8 | 89 |  | 11 |  |  |  | 43 |  |  |  | 17 |  |  |  | 5 |  |  |  |  |  |  |  |  |  |  |  |  |  |  |  | 33 |  |  |  |  |  |  |  |  |  |  |  |  |  |  |  | 12 |  |  |  |  |  |  |  |  |  |  |  |  |  |  |
| AAG | 78 | 21 | 3 | 16 |  |  | 16 |  |  |  | 35 |  |  |  | 19 |  |  |  | 14 |  |  |  |  |  |  |  |  |  |  |  |  |  |  |  | 24 |  |  |  |  |  |  |  |  |  |  |  |  |  |  |  | 8 |  |  |  |  |  |  |  |  |  |  |  |  |  |
| AAT | 5 | 56 | 9 | 4 |  |  |  | 18 |  |  |  | 14 |  |  |  | 11 |  |  |  | 2 |  |  |  |  |  |  |  |  |  |  |  |  |  |  |  | 25 |  |  |  |  |  |  |  |  |  |  |  |  |  |  |  | 13 |  |  |  |  |  |  |  |  |  |  |  |  |
| ACA | 22 |  |  |  | 5 | 12 | 87 | 24 | 13 |  |  |  | 29 |  |  |  |  |  |  |  | 4 |  |  |  |  |  |  |  |  |  |  |  |  |  |  |  | 34 |  |  |  |  |  |  |  |  |  |  |  |  |  |  |  | 13 |  |  |  |  |  |  |  |  |  |  |  |
| ACC |  | 13 |  |  | 17 | 1 | 27 | 53 |  | 7 |  |  |  | 29 |  |  |  |  |  |  |  | 1 |  |  |  |  |  |  |  |  |  |  |  |  |  |  |  | 37 |  |  |  |  |  |  |  |  |  |  |  |  |  |  |  | 10 |  |  |  |  |  |  |  |  |  |  |
| ACG |  |  | 7 |  | 66 | 13 |  | 22 |  |  | 9 |  |  |  | 50 |  |  |  |  |  |  |  | 7 |  |  |  |  |  |  |  |  |  |  |  |  |  |  |  | 20 |  |  |  |  |  |  |  |  |  |  |  |  |  |  |  | 11 |  |  |  |  |  |  |  |  |  |
| ACT |  |  |  | 19 | 20 | 57 | 15 | 3 |  |  |  | 12 |  |  |  | 21 |  |  |  |  |  |  |  | 18 |  |  |  |  |  |  |  |  |  |  |  |  |  |  |  | 34 |  |  |  |  |  |  |  |  |  |  |  |  |  |  |  | 10 |  |  |  |  |  |  |  |  |
| AGA | 22 |  |  |  | 8 |  |  |  | 3 | 3 | 35 | 12 | 6 |  |  |  |  |  |  |  |  |  |  |  | 17 |  |  |  |  |  |  |  |  |  |  |  |  |  |  |  | 16 |  |  |  |  |  |  |  |  |  |  |  |  |  |  |  | 1 |  |  |  |  |  |  |  |
| AGC |  | 43 |  |  |  | 30 |  |  | 19 | 7 | 13 | 97 |  | 24 |  |  |  |  |  |  |  |  |  |  |  | 12 |  |  |  |  |  |  |  |  |  |  |  |  |  |  |  | 34 |  |  |  |  |  |  |  |  |  |  |  |  |  |  |  | 8 |  |  |  |  |  |  |
| AGG |  |  | 47 |  |  |  | 8 |  | 64 | 9 | 2 | 11 |  |  | 13 |  |  |  |  |  |  |  |  |  |  |  | 13 |  |  |  |  |  |  |  |  |  |  |  |  |  |  |  | 19 |  |  |  |  |  |  |  |  |  |  |  |  |  |  |  | 6 |  |  |  |  |  |
| AGT |  |  |  | 48 |  |  |  | 15 | 5 | 64 | 5 | 9 |  |  |  | 10 |  |  |  |  |  |  |  |  |  |  |  | 4 |  |  |  |  |  |  |  |  |  |  |  |  |  |  |  | 21 |  |  |  |  |  |  |  |  |  |  |  |  |  |  |  | 10 |  |  |  |  |
| ATA | 5 |  |  |  | 16 |  |  |  | 7 |  |  |  | 1 | 14 | 17 | 25 |  |  |  |  |  |  |  |  |  |  |  |  | 2 |  |  |  |  |  |  |  |  |  |  |  |  |  |  |  | 26 |  |  |  |  |  |  |  |  |  |  |  |  |  |  |  | 10 |  |  |  |
| ATC |  | 7 |  |  |  | 11 |  |  |  | 5 |  |  | 35 | 5 | 4 | 54 |  |  |  |  |  |  |  |  |  |  |  |  |  | 5 |  |  |  |  |  |  |  |  |  |  |  |  |  |  |  | 25 |  |  |  |  |  |  |  |  |  |  |  |  |  |  |  | 17 |  |  |
| ATG |  |  | 4 |  |  |  | 52 |  |  |  | 9 |  | 28 | 12 | 5 | 14 |  |  |  |  |  |  |  |  |  |  |  |  |  |  | 10 |  |  |  |  |  |  |  |  |  |  |  |  |  |  |  | 41 |  |  |  |  |  |  |  |  |  |  |  |  |  |  |  | 29 |  |
| ATT |  |  |  | 10 |  |  |  | 17 |  |  |  | 14 | 15 | 61 |  | 1 |  |  |  |  |  |  |  |  |  |  |  |  |  |  |  | 6 |  |  |  |  |  |  |  |  |  |  |  |  |  |  |  | 48 |  |  |  |  |  |  |  |  |  |  |  |  |  |  |  | 7 |
| CAA | 16 |  |  |  |  |  |  |  |  |  |  |  |  |  |  |  | 3 | 12 | 62 | 12 |  |  |  |  | 28 |  |  |  | 11 |  |  |  | 29 |  |  |  |  |  |  |  |  |  |  |  |  |  |  |  | 7 |  |  |  |  |  |  |  |  |  |  |  |  |  |  |  |
| CAC |  | 13 |  |  |  |  |  |  |  |  |  |  |  |  |  |  | 5 | 1 | 12 | 50 |  | 6 |  |  |  | 17 |  |  |  | 9 |  |  |  | 5 |  |  |  |  |  |  |  |  |  |  |  |  |  |  |  | 11 |  |  |  |  |  |  |  |  |  |  |  |  |  |  |
| CAG |  |  | 41 |  |  |  |  |  |  |  |  |  |  |  |  |  | 100 | 11 | 4 | 35 |  |  | 11 |  |  |  | 52 |  |  |  | 35 |  |  |  | 21 |  |  |  |  |  |  |  |  |  |  |  |  |  |  |  | 6 |  |  |  |  |  |  |  |  |  |  |  |  |  |
| CAT |  |  |  | 12 |  |  |  |  |  |  |  |  |  |  |  |  | 8 | 46 | 7 | 4 |  |  |  | 1 |  |  |  | 33 |  |  |  | 5 |  |  |  | 10 |  |  |  |  |  |  |  |  |  |  |  |  |  |  |  | 16 |  |  |  |  |  |  |  |  |  |  |  |  |
| CCA |  |  |  |  | 7 |  |  |  |  |  |  |  |  |  |  |  | 9 |  |  |  | 3 | 12 | 65 | 30 | 11 |  |  |  | 12 |  |  |  |  |  |  |  | 12 |  |  |  |  |  |  |  |  |  |  |  |  |  |  |  | 28 |  |  |  |  |  |  |  |  |  |  |  |
| CCC |  |  |  |  |  | 5 |  |  |  |  |  |  |  |  |  |  |  | 9 |  |  | 17 | 3 | 16 | 50 |  | 3 |  |  |  | 28 |  |  |  |  |  |  |  | 10 |  |  |  |  |  |  |  |  |  |  |  |  |  |  |  | 22 |  |  |  |  |  |  |  |  |  |  |
| CCG |  |  |  |  |  |  | 12 |  |  |  |  |  |  |  |  |  |  |  | 18 |  | 110 | 15 | 4 | 46 |  |  | 3 |  |  |  | 43 |  |  |  |  |  |  |  | 6 |  |  |  |  |  |  |  |  |  |  |  |  |  |  |  | 17 |  |  |  |  |  |  |  |  |  |
| CCT |  |  |  |  |  |  |  | 16 |  |  |  |  |  |  |  |  |  |  |  | 29 | 22 | 43 | 16 | 1 |  |  |  | 12 |  |  |  | 21 |  |  |  |  |  |  |  | 18 |  |  |  |  |  |  |  |  |  |  |  |  |  |  |  | 35 |  |  |  |  |  |  |  |  |
| CGA |  |  |  |  |  |  |  |  | 13 |  |  |  |  |  |  |  | 43 |  |  |  | 8 |  |  |  | 7 | 10 | 17 | 5 | 14 |  |  |  |  |  |  |  |  |  |  |  | 4 |  |  |  |  |  |  |  |  |  |  |  |  |  |  |  | 12 |  |  |  |  |  |  |  |
| CGC |  |  |  |  |  |  |  |  |  | 14 |  |  |  |  |  |  |  | 30 |  |  |  | 4 |  |  | 21 | 3 | 17 | 31 |  | 16 |  |  |  |  |  |  |  |  |  |  |  | 5 |  |  |  |  |  |  |  |  |  |  |  |  |  |  |  | 28 |  |  |  |  |  |  |
| CGG |  |  |  |  |  |  |  |  |  |  | 12 |  |  |  |  |  |  |  | 36 |  |  |  | 4 |  | 19 | 14 | 2 | 15 |  |  | 16 |  |  |  |  |  |  |  |  |  |  |  | 10 |  |  |  |  |  |  |  |  |  |  |  |  |  |  |  | 21 |  |  |  |  |  |
| CGT |  |  |  |  |  |  |  |  |  |  |  | 6 |  |  |  |  |  |  |  | 25 |  |  |  |  | 16 | 27 | 2 |  |  |  |  | 5 |  |  |  |  |  |  |  |  |  |  |  | 5 |  |  |  |  |  |  |  |  |  |  |  |  |  |  |  | 23 |  |  |  |  |
| CTA |  |  |  |  |  |  |  |  |  |  |  |  | 4 |  |  |  | 3 |  |  |  | 2 |  |  |  | 5 |  |  |  | 2 | 13 | 48 | 10 |  |  |  |  |  |  |  |  |  |  |  |  | 4 |  |  |  |  |  |  |  |  |  |  |  |  |  |  |  | 9 |  |  |  |
| CTC |  |  |  |  |  |  |  |  |  |  |  |  |  | 17 |  |  |  | 19 |  |  |  | 4 |  |  |  | 4 |  |  | 34 | 6 | 39 | 76 |  |  |  |  |  |  |  |  |  |  |  |  |  | 13 |  |  |  |  |  |  |  |  |  |  |  |  |  |  |  | 30 |  |  |
| CTG |  |  |  |  |  |  |  |  |  |  |  |  |  |  | 26 |  |  |  | 12 |  |  |  | 32 |  |  |  | 5 |  | 98 | 42 | 11 | 25 |  |  |  |  |  |  |  |  |  |  |  |  |  |  | 14 |  |  |  |  |  |  |  |  |  |  |  |  |  |  |  | 106 |  |
| CTT |  |  |  |  |  |  |  |  |  |  |  |  |  |  |  | 19 |  |  |  | 16 |  |  |  | 5 |  |  |  | 9 | 21 | 47 | 10 | 7 |  |  |  |  |  |  |  |  |  |  |  |  |  |  |  | 18 |  |  |  |  |  |  |  |  |  |  |  |  |  |  |  | 15 |
| GAA | 47 |  |  |  |  |  |  |  |  |  |  |  |  |  |  |  | 19 |  |  |  |  |  |  |  |  |  |  |  |  |  |  |  | 4 | 14 | 58 | 36 | 16 |  |  |  | 19 |  |  |  | 10 |  |  |  | 6 |  |  |  |  |  |  |  |  |  |  |  |  |  |  |  |
| GAC |  | 34 |  |  |  |  |  |  |  |  |  |  |  |  |  |  |  | 6 |  |  |  |  |  |  |  |  |  |  |  |  |  |  | 14 | 3 | 29 | 142 |  | 4 |  |  |  | 15 |  |  |  | 4 |  |  |  | 8 |  |  |  |  |  |  |  |  |  |  |  |  |  |  |
| GAG |  |  | 42 |  |  |  |  |  |  |  |  |  |  |  |  |  |  |  | 23 |  |  |  |  |  |  |  |  |  |  |  |  |  | 86 | 33 | 4 | 34 |  |  | 28 |  |  |  | 24 |  |  |  | 21 |  |  |  | 6 |  |  |  |  |  |  |  |  |  |  |  |  |  |
| GAT |  |  |  | 32 |  |  |  |  |  |  |  |  |  |  |  |  |  |  |  | 8 |  |  |  |  |  |  |  |  |  |  |  |  | 43 | 104 | 13 | 10 |  |  |  | 11 |  |  |  | 14 |  |  |  | 11 |  |  |  | 16 |  |  |  |  |  |  |  |  |  |  |  |  |
| GCA |  |  |  |  | 62 |  |  |  |  |  |  |  |  |  |  |  |  |  |  |  | 12 |  |  |  |  |  |  |  |  |  |  |  | 26 |  |  |  | 2 | 25 | 83 | 74 | 16 |  |  |  | 34 |  |  |  |  |  |  |  | 30 |  |  |  |  |  |  |  |  |  |  |  |
| GCC |  |  |  |  |  | 69 |  |  |  |  |  |  |  |  |  |  |  |  |  |  |  | 15 |  |  |  |  |  |  |  |  |  |  |  | 21 |  |  | 35 | 2 | 19 | 123 |  | 22 |  |  |  | 48 |  |  |  |  |  |  |  | 18 |  |  |  |  |  |  |  |  |  |  |
| GCG |  |  |  |  |  |  | 38 |  |  |  |  |  |  |  |  |  |  |  |  |  |  |  | 11 |  |  |  |  |  |  |  |  |  |  |  | 21 |  | 113 | 33 | 4 | 26 |  |  | 12 |  |  |  | 41 |  |  |  |  |  |  |  | 10 |  |  |  |  |  |  |  |  |  |
| GCT |  |  |  |  |  |  |  | 56 |  |  |  |  |  |  |  |  |  |  |  |  |  |  |  | 40 |  |  |  |  |  |  |  |  |  |  |  | 24 | 62 | 82 | 42 | 5 |  |  |  | 17 |  |  |  | 50 |  |  |  |  |  |  |  | 46 |  |  |  |  |  |  |  |  |
| GGA |  |  |  |  |  |  |  |  | 46 |  |  |  |  |  |  |  |  |  |  |  |  |  |  |  | 9 |  |  |  |  |  |  |  | 22 |  |  |  | 15 |  |  |  | 5 | 8 | 34 | 24 | 7 |  |  |  |  |  |  |  |  |  |  |  | 5 |  |  |  |  |  |  |  |
| GGC |  |  |  |  |  |  |  |  |  | 32 |  |  |  |  |  |  |  |  |  |  |  |  |  |  |  | 6 |  |  |  |  |  |  |  | 31 |  |  |  | 10 |  |  | 31 |  | 18 | 99 |  | 11 |  |  |  |  |  |  |  |  |  |  |  | 14 |  |  |  |  |  |  |
| GGG |  |  |  |  |  |  |  |  |  |  | 17 |  |  |  |  |  |  |  |  |  |  |  |  |  |  |  | 8 |  |  |  |  |  |  |  | 19 |  |  |  | 16 |  | 58 | 21 |  | 31 |  |  | 4 |  |  |  |  |  |  |  |  |  |  |  | 3 |  |  |  |  |  |
| GGT |  |  |  |  |  |  |  |  |  |  |  | 32 |  |  |  |  |  |  |  |  |  |  |  |  |  |  |  | 4 |  |  |  |  |  |  |  | 23 |  |  |  | 8 | 30 | 44 | 12 | 2 |  |  |  | 11 |  |  |  |  |  |  |  |  |  |  |  | 13 |  |  |  |  |
| GTA |  |  |  |  |  |  |  |  |  |  |  |  | 30 |  |  |  |  |  |  |  |  |  |  |  |  |  |  |  | 1 |  |  |  | 3 |  |  |  | 15 |  |  |  | 4 |  |  |  | 1 | 27 | 25 | 10 |  |  |  |  |  |  |  |  |  |  |  |  | 20 |  |  |  |
| GTC |  |  |  |  |  |  |  |  |  |  |  |  |  | 58 |  |  |  |  |  |  |  |  |  |  |  |  |  |  |  | 17 |  |  |  | 14 |  |  |  | 22 |  |  |  | 11 |  |  | 22 | 4 | 33 | 77 |  |  |  |  |  |  |  |  |  |  |  |  |  | 4 |  |  |
| GTG |  |  |  |  |  |  |  |  |  |  |  |  |  |  | 57 |  |  |  |  |  |  |  |  |  |  |  |  |  |  |  | 8 |  |  |  | 11 |  |  |  | 51 |  |  |  | 10 |  | 50 | 25 | 1 | 20 |  |  |  |  |  |  |  |  |  |  |  |  |  |  | 14 |  |
| GTT |  |  |  |  |  |  |  |  |  |  |  |  |  |  |  | 73 |  |  |  |  |  |  |  |  |  |  |  |  |  |  |  | 8 |  |  |  | 13 |  |  |  | 33 |  |  |  | 7 | 30 | 49 | 32 |  |  |  |  |  |  |  |  |  |  |  |  |  |  |  |  | 8 |
| TAA | 1 |  |  |  |  |  |  |  |  |  |  |  |  |  |  |  | 3 |  |  |  |  |  |  |  |  |  |  |  |  |  |  |  |  |  |  |  |  |  |  |  |  |  |  |  |  |  |  |  | 2 | 1 | 1 | 2 | 3 |  |  |  | 1 |  |  |  |  |  |  |  |
| TAC |  | 3 |  |  |  |  |  |  |  |  |  |  |  |  |  |  |  | 18 |  |  |  |  |  |  |  |  |  |  |  |  |  |  |  | 9 |  |  |  |  |  |  |  |  |  |  |  |  |  |  |  | 2 | 8 | 55 |  | 6 |  |  |  | 21 |  |  |  | 13 |  |  |
| TAG |  |  |  |  |  |  |  |  |  |  |  |  |  |  |  |  |  |  | 1 |  |  |  |  |  |  |  |  |  |  |  |  |  |  |  | 3 |  |  |  |  |  |  |  |  |  |  |  |  |  | 1 | 1 | 4 |  |  |  |  |  |  |  | 5 |  |  |  | 2 |  |
| TAT |  |  |  | 9 |  |  |  |  |  |  |  |  |  |  |  |  |  |  |  | 15 |  |  |  |  |  |  |  |  |  |  |  |  |  |  |  | 1 |  |  |  |  |  |  |  |  |  |  |  |  | 1 | 44 | 2 | 1 |  |  |  | 7 |  |  |  | 5 |  |  |  | 18 |
| TCA |  |  |  |  | 7 |  |  |  |  |  |  |  |  |  |  |  |  |  |  |  | 21 |  |  |  |  |  |  |  |  |  |  |  |  |  |  |  | 36 |  |  |  |  |  |  |  |  |  |  |  | 2 |  |  |  | 5 | 5 | 108 | 34 | 9 |  |  |  | 18 |  |  |  |
| TCC |  |  |  |  |  | 15 |  |  |  |  |  |  |  |  |  |  |  |  |  |  |  | 26 |  |  |  |  |  |  |  |  |  |  |  |  |  |  |  | 17 |  |  |  |  |  |  |  |  |  |  |  | 14 |  |  | 27 | 7 | 32 | 89 |  | 9 |  |  |  | 6 |  |  |
| TCG |  |  |  |  |  |  | 11 |  |  |  |  |  |  |  |  |  |  |  |  |  |  |  | 21 |  |  |  |  |  |  |  |  |  |  |  |  |  |  |  | 11 |  |  |  |  |  |  |  |  |  |  |  | 3 |  | 123 | 21 | 2 | 26 |  |  | 5 |  |  |  | 38 |  |
| TCT |  |  |  |  |  |  |  | 14 |  |  |  |  |  |  |  |  |  |  |  |  |  |  |  | 27 |  |  |  |  |  |  |  |  |  |  |  |  |  |  |  | 23 |  |  |  |  |  |  |  |  |  |  |  | 16 | 20 | 37 | 20 | 2 |  |  |  | 11 |  |  |  | 18 |
| TGA |  |  |  |  |  |  |  |  | 5 |  |  |  |  |  |  |  |  |  |  |  |  |  |  |  | 1 |  |  |  |  |  |  |  |  |  |  |  |  |  |  |  | 7 |  |  |  |  |  |  |  | 2 |  |  |  | 9 |  |  |  | 6 | 1 | 2 | 4 |  |  |  |  |
| TGC |  |  |  |  |  |  |  |  |  | 9 |  |  |  |  |  |  |  |  |  |  |  |  |  |  |  | 7 |  |  |  |  |  |  |  |  |  |  |  |  |  |  |  | 7 |  |  |  |  |  |  |  | 12 |  |  |  | 13 |  |  | 3 | 5 | 3 | 58 |  | 5 |  |  |
| TGG |  |  |  |  |  |  |  |  |  |  | 4 |  |  |  |  |  |  |  |  |  |  |  |  |  |  |  | 8 |  |  |  |  |  |  |  |  |  |  |  |  |  |  |  | 4 |  |  |  |  |  |  |  | 5 |  |  |  | 5 |  | 11 | 4 | 3 | 5 |  |  | 4 |  |
| TGT |  |  |  |  |  |  |  |  |  |  |  | 11 |  |  |  |  |  |  |  |  |  |  |  |  |  |  |  | 10 |  |  |  |  |  |  |  |  |  |  |  |  |  |  |  | 2 |  |  |  |  |  |  |  | 12 |  |  |  | 5 | 2 | 21 | 2 | 3 |  |  |  | 3 |
| TTA |  |  |  |  |  |  |  |  |  |  |  |  | 3 |  |  |  |  |  |  |  |  |  |  |  |  |  |  |  | 13 |  |  |  |  |  |  |  |  |  |  |  |  |  |  |  | 6 |  |  |  |  |  |  |  | 10 |  |  |  | 2 |  |  |  |  | 3 | 30 | 9 |
| TTC |  |  |  |  |  |  |  |  |  |  |  |  |  | 11 |  |  |  |  |  |  |  |  |  |  |  |  |  |  |  | 13 |  |  |  |  |  |  |  |  |  |  |  |  |  |  |  | 8 |  |  |  | 15 |  |  |  | 17 |  |  |  | 4 |  |  | 14 | 7 | 4 | 64 |
| TTG |  |  |  |  |  |  |  |  |  |  |  |  |  |  | 9 |  |  |  |  |  |  |  |  |  |  |  |  |  |  |  | 62 |  |  |  |  |  |  |  |  |  |  |  |  |  |  |  | 9 |  |  |  | 3 |  |  |  | 36 |  |  |  | 2 |  | 35 | 11 | 2 | 6 |
| TTT |  |  |  |  |  |  |  |  |  |  |  |  |  |  |  | 9 |  |  |  |  |  |  |  |  |  |  |  |  |  |  |  | 16 |  |  |  |  |  |  |  |  |  |  |  |  |  |  |  | 4 |  |  |  | 14 |  |  |  | 6 |  |  |  | 2 | 9 | 41 | 4 | 2 |


---


 **Amino acid changes**

How to read this table:   
- Rows are reference amino acids and columns are changed amino acids. E.g. Row 'A' column 'E' indicates how many 'A' amino acids have been replaced by 'E' amino acids.  
- Red background colors indicate that more changes happened (heat-map).  
- Diagonals are indicated using grey background color   
- WARNING: This table may include different translation codon tables (e.g. mamalian DNA and mitochondrial DNA).

|  | \* | ? | A | C | D | E | F | G | H | I | K | L | M | N | P | Q | R | S | T | V | W | Y |
| --- | --- | --- | --- | --- | --- | --- | --- | --- | --- | --- | --- | --- | --- | --- | --- | --- | --- | --- | --- | --- | --- | --- |
| \* | 17 |  |  | 5 |  | 3 |  | 7 |  |  | 1 | 2 |  |  |  | 4 | 6 | 12 |  |  | 7 | 4 |
| ? |  | 2 |  |  |  |  |  |  |  |  |  |  |  |  |  |  |  |  |  |  |  |  |
| A |  |  | 730 |  | 45 | 47 |  | 67 |  |  |  |  |  |  | 78 |  |  | 104 | 225 | 173 |  |  |
| C | 5 |  |  | 87 |  |  | 8 | 9 |  |  |  |  |  |  |  |  | 17 | 38 |  |  | 5 | 24 |
| D |  |  | 15 |  | 259 | 99 |  | 29 | 14 |  |  |  |  | 66 |  |  |  |  |  | 15 |  | 24 |
| E | 12 |  | 44 |  | 117 | 152 |  | 43 |  |  | 89 |  |  |  |  | 42 |  |  |  | 31 |  |  |
| F |  |  |  | 6 |  |  | 114 |  |  | 20 |  | 60 |  |  |  |  |  | 23 |  | 12 |  | 29 |
| G | 5 |  | 49 | 27 | 54 | 41 |  | 417 |  |  |  |  |  |  |  |  | 90 | 64 |  | 33 | 3 |  |
| H |  |  |  |  | 15 |  |  |  | 101 |  |  | 14 |  | 25 | 7 | 32 | 50 |  |  |  |  | 27 |
| I |  |  |  |  |  |  | 24 |  |  | 211 | 5 | 23 | 21 | 17 |  |  | 7 | 19 | 44 | 99 |  |  |
| K | 12 |  |  |  |  | 51 |  |  |  | 18 | 131 |  | 19 | 67 |  | 31 | 57 |  | 29 |  |  |  |
| L | 5 |  |  |  |  |  | 74 |  | 35 | 43 |  | 746 | 35 |  | 43 | 15 | 23 | 46 |  | 64 | 2 |  |
| M |  |  |  |  |  |  |  |  |  | 54 | 4 | 39 | 5 |  |  |  | 9 |  | 52 | 41 |  |  |
| N |  |  |  |  | 58 |  |  |  | 7 | 28 | 31 |  |  | 150 |  |  |  | 57 | 29 |  |  | 25 |
| P |  |  | 46 |  |  |  |  |  | 38 |  |  | 104 |  |  | 453 | 27 | 29 | 102 | 40 |  |  |  |
| Q | 13 |  |  |  |  | 50 |  |  | 70 |  | 57 | 46 |  |  | 11 | 169 | 80 |  |  |  |  |  |
| R | 13 |  |  | 51 |  |  |  | 59 | 55 | 6 | 69 | 51 | 13 |  | 16 | 79 | 365 | 55 | 16 |  | 27 |  |
| S | 14 |  | 87 | 38 |  |  | 24 | 55 |  | 34 |  | 56 |  | 91 | 95 |  | 58 | 735 | 92 |  | 5 | 30 |
| T |  |  | 125 |  |  |  |  |  |  | 79 | 29 |  | 50 | 32 | 30 |  | 22 | 63 | 422 |  |  |  |
| V |  |  | 121 |  | 27 | 14 | 12 | 32 |  | 161 |  | 68 | 57 |  |  |  |  |  |  | 406 |  |  |
| W | 16 |  |  | 9 |  |  |  | 4 |  |  |  | 4 |  |  |  |  | 12 | 5 |  |  | 3 |  |
| Y | 11 |  |  | 26 | 10 |  | 31 |  | 33 |  |  |  |  | 12 |  |  |  | 13 |  |  |  | 102 |


---


 **Variants by chromosome**

```
		  

		1, Position,0,100000,200000,300000,400000,500000,600000,700000,800000,900000,1000000,1100000,1200000,1300000,1400000,1500000,1600000,1700000,1800000,1900000,2000000,2100000,2200000,2300000,2400000,2500000,2600000,2700000,2800000,2900000,3000000,3100000,3200000,3300000,3400000,3500000,3600000,3700000,3800000,3900000,4000000,4100000,4200000,4300000,4400000,4500000,4600000,4700000,4800000,4900000,5000000,5100000,5200000,5300000,5400000,5500000,5600000,5700000,5800000,5900000,6000000,6100000,6200000,6300000,6400000,6500000,6600000,6700000,6800000,6900000,7000000,7100000,7200000,7300000,7400000,7500000,7600000,7700000,7800000,7900000,8000000,8100000,8200000,8300000,8400000,8500000,8600000,8700000,8800000,8900000,9000000,9100000,9200000,9300000,9400000,9500000,9600000,9700000,9800000,9900000,10000000,10100000,10200000,10300000,10400000,10500000,10600000,10700000,10800000,10900000,11000000,11100000,11200000,11300000,11400000,11500000,11600000,11700000,11800000,11900000,12000000,12100000,12200000,12300000,12400000,12500000,12600000,12700000,12800000,12900000,13000000,13100000,13200000,13300000,13400000,13500000,13600000,13700000,13800000,13900000,14000000,14100000,14200000,14300000,14400000,14500000,14600000,14700000,14800000,14900000,15000000,15100000,15200000,15300000,15400000,15500000,15600000,15700000,15800000,15900000,16000000,16100000,16200000,16300000,16400000,16500000,16600000,16700000,16800000,16900000,17000000,17100000,17200000,17300000,17400000,17500000,17600000,17700000,17800000,17900000,18000000,18100000,18200000,18300000,18400000,18500000,18600000,18700000,18800000,18900000,19000000,19100000,19200000,19300000,19400000,19500000,19600000,19700000,19800000,19900000,20000000,20100000,20200000,20300000,20400000,20500000,20600000,20700000,20800000,20900000,21000000,21100000,21200000,21300000,21400000,21500000,21600000,21700000,21800000,21900000,22000000,22100000,22200000,22300000,22400000,22500000,22600000,22700000,22800000,22900000,23000000,23100000,23200000,23300000,23400000,23500000,23600000,23700000,23800000,23900000,24000000,24100000,24200000,24300000,24400000,24500000,24600000,24700000,24800000,24900000,25000000,25100000,25200000,25300000,25400000,25500000,25600000,25700000,25800000,25900000,26000000,26100000,26200000,26300000,26400000,26500000,26600000,26700000,26800000,26900000,27000000,27100000,27200000,27300000,27400000,27500000,27600000,27700000,27800000,27900000,28000000,28100000,28200000,28300000,28400000,28500000,28600000,28700000,28800000,28900000,29000000
1,Count,3,19,0,16,21,10,7,7,10,5,13,44,22,2,23,0,10,9,26,4,8,17,5,31,12,0,4,7,0,7,8,15,6,12,6,18,0,6,8,19,35,25,6,5,5,34,0,10,4,3,6,7,13,9,7,14,10,31,12,2,9,1,0,8,4,16,6,1,5,15,0,3,5,5,23,26,15,23,6,1,14,10,5,9,2,9,9,2,14,18,44,0,6,11,5,5,6,5,23,5,1,0,6,19,9,7,14,20,6,27,8,5,18,11,39,14,19,11,14,28,13,8,7,16,17,6,1,18,1,12,3,25,3,6,6,29,9,7,3,12,20,2,5,3,2,14,3,0,0,0,5,32,0,4,4,4,0,0,0,0,0,0,0,0,10,20,0,0,0,0,15,0,0,4,0,6,4,0,5,0,10,0,0,0,0,0,0,0,32,0,10,2,0,0,0,0,10,4,0,0,0,0,4,0,0,0,0,0,0,0,4,0,0,0,3,0,6,1,6,0,0,6,19,8,0,0,0,0,0,0,0,0,0,0,0,0,0,0,0,1,0,0,0,0,0,0,0,0,0,4,0,1,0,25,9,0,0,5,0,16,6,5,8,0,3,20,0,11,0,0,0,0,0,0,15,4,0,6,14,0,4,0,4,0,2,0,0,13,0,8,0

	
```

```
		  

		2, Position,0,100000,200000,300000,400000,500000,600000,700000,800000,900000,1000000,1100000,1200000,1300000,1400000,1500000,1600000,1700000,1800000,1900000,2000000,2100000,2200000,2300000,2400000,2500000,2600000,2700000,2800000,2900000,3000000,3100000,3200000,3300000,3400000,3500000,3600000,3700000,3800000,3900000,4000000,4100000,4200000,4300000,4400000,4500000,4600000,4700000,4800000,4900000,5000000,5100000,5200000,5300000,5400000,5500000,5600000,5700000,5800000,5900000,6000000,6100000,6200000,6300000,6400000,6500000,6600000,6700000,6800000,6900000,7000000,7100000,7200000,7300000,7400000,7500000,7600000,7700000,7800000,7900000,8000000,8100000,8200000,8300000,8400000,8500000,8600000,8700000,8800000,8900000,9000000,9100000,9200000,9300000,9400000,9500000,9600000,9700000,9800000,9900000,10000000,10100000,10200000,10300000,10400000,10500000,10600000,10700000,10800000,10900000,11000000,11100000,11200000,11300000,11400000,11500000,11600000,11700000,11800000,11900000,12000000,12100000,12200000,12300000,12400000,12500000,12600000,12700000,12800000,12900000,13000000,13100000,13200000,13300000,13400000,13500000,13600000,13700000,13800000,13900000,14000000,14100000,14200000,14300000,14400000,14500000,14600000,14700000,14800000,14900000,15000000,15100000,15200000,15300000,15400000,15500000,15600000,15700000,15800000,15900000,16000000,16100000,16200000,16300000,16400000,16500000,16600000,16700000,16800000,16900000,17000000,17100000,17200000,17300000,17400000,17500000,17600000,17700000,17800000,17900000,18000000,18100000,18200000,18300000,18400000,18500000,18600000,18700000,18800000,18900000,19000000,19100000,19200000,19300000,19400000,19500000,19600000,19700000,19800000,19900000,20000000,20100000,20200000,20300000,20400000,20500000,20600000,20700000,20800000,20900000,21000000,21100000,21200000,21300000,21400000,21500000,21600000,21700000,21800000,21900000,22000000,22100000,22200000,22300000,22400000,22500000,22600000,22700000,22800000,22900000,23000000,23100000,23200000,23300000,23400000,23500000,23600000,23700000,23800000,23900000,24000000,24100000,24200000,24300000,24400000,24500000,24600000,24700000,24800000,24900000,25000000,25100000,25200000,25300000,25400000,25500000,25600000,25700000,25800000,25900000,26000000,26100000,26200000,26300000,26400000,26500000,26600000,26700000,26800000,26900000,27000000,27100000,27200000,27300000,27400000,27500000,27600000,27700000,27800000,27900000,28000000,28100000,28200000,28300000,28400000,28500000,28600000,28700000,28800000,28900000,29000000,29100000,29200000,29300000,29400000,29500000
2,Count,1,0,0,0,0,0,0,0,0,0,5,0,0,0,0,0,0,0,0,0,0,0,1,0,26,0,0,6,0,0,0,0,0,0,0,0,0,0,0,0,0,0,0,0,0,7,0,0,0,0,0,0,0,0,21,0,0,0,0,0,0,0,8,0,0,0,2,0,1,0,0,2,7,12,0,0,0,0,0,0,0,3,0,0,0,1,0,0,0,0,0,0,0,0,0,0,1,0,2,11,10,0,0,4,0,8,4,0,0,5,0,0,0,0,0,0,0,0,5,0,0,5,0,0,0,0,0,19,0,2,11,3,0,8,10,0,1,0,0,9,0,1,4,18,10,0,25,3,9,0,13,18,0,7,0,3,3,7,25,10,9,5,5,12,10,9,0,0,0,27,5,0,3,5,0,10,7,1,1,7,19,21,7,21,9,22,2,5,8,4,20,0,14,10,12,4,25,0,1,12,11,3,7,14,16,2,6,1,23,1,2,3,4,0,8,10,19,5,15,0,0,4,9,53,11,19,0,32,12,14,22,2,19,8,14,4,9,10,8,6,14,13,19,11,12,9,11,1,5,12,12,20,14,5,9,5,11,0,8,3,16,8,0,11,15,11,10,11,6,5,0,10,14,10,2,18,16,4,0,1,11,2,7,2,11,23,26,9,21,6,22,16,0,5,0,0

	
```

```
		  

		3, Position,0,1000000,2000000,3000000,4000000,5000000,6000000,7000000,8000000,9000000,10000000,11000000,12000000,13000000,14000000,15000000,16000000,17000000,18000000,19000000,20000000,21000000,22000000,23000000,24000000,25000000,26000000,27000000,28000000,29000000,30000000,31000000,32000000,33000000,34000000,35000000
3,Count,117,155,111,105,142,57,101,137,69,79,44,21,5,45,43,27,4,38,46,22,35,12,27,43,90,108,83,65,132,137,122,91,72,115,108,0

	
```

```
		  

		4, Position,0,1000000,2000000,3000000,4000000,5000000,6000000,7000000,8000000,9000000,10000000,11000000,12000000,13000000,14000000,15000000,16000000,17000000,18000000,19000000,20000000,21000000,22000000,23000000,24000000,25000000,26000000,27000000,28000000,29000000,30000000,31000000,32000000,33000000,34000000,35000000,36000000,37000000
4,Count,75,76,76,78,85,116,154,131,67,70,103,84,5,14,13,17,4,34,18,28,40,14,96,82,113,86,97,105,65,79,150,149,108,131,177,89,115,2

	
```

```
		  

		5, Position,0,1000000,2000000,3000000,4000000,5000000,6000000,7000000,8000000,9000000,10000000,11000000,12000000,13000000,14000000,15000000,16000000,17000000,18000000,19000000,20000000,21000000,22000000,23000000,24000000,25000000,26000000,27000000,28000000,29000000,30000000,31000000,32000000,33000000,34000000,35000000,36000000,37000000,38000000,39000000,40000000,41000000
5,Count,131,79,62,78,116,61,154,75,111,86,101,40,6,0,5,26,22,16,17,78,14,41,22,26,22,30,3,15,10,0,5,13,78,62,41,42,86,156,70,136,121,105

	
```

```
		  

		6, Position,0,1000000,2000000,3000000,4000000,5000000,6000000,7000000,8000000,9000000,10000000,11000000,12000000,13000000,14000000,15000000,16000000,17000000,18000000,19000000,20000000,21000000,22000000,23000000,24000000,25000000,26000000,27000000,28000000,29000000,30000000,31000000,32000000,33000000,34000000,35000000,36000000,37000000
6,Count,98,140,151,80,86,120,235,116,104,106,109,140,114,96,33,81,17,85,11,22,28,23,35,9,16,70,24,4,57,109,142,133,138,87,115,145,97,54

	
```

```
		  

		7, Position,0,1000000,2000000,3000000,4000000,5000000,6000000,7000000,8000000,9000000,10000000,11000000,12000000,13000000,14000000,15000000,16000000,17000000,18000000,19000000,20000000,21000000,22000000,23000000,24000000,25000000,26000000,27000000,28000000,29000000,30000000,31000000,32000000,33000000,34000000,35000000
7,Count,134,88,131,183,106,73,129,136,81,69,123,62,22,5,0,36,32,8,0,0,11,10,11,0,15,8,21,104,53,48,177,175,125,131,136,0

	
```

```
		  

		8, Position,0,1000000,2000000,3000000,4000000,5000000,6000000,7000000,8000000,9000000,10000000,11000000,12000000,13000000,14000000,15000000,16000000,17000000,18000000,19000000,20000000,21000000,22000000,23000000,24000000,25000000,26000000,27000000,28000000,29000000,30000000,31000000,32000000,33000000,34000000,35000000,36000000,37000000,38000000,39000000,40000000,41000000,42000000,43000000,44000000
8,Count,74,84,136,114,107,134,66,99,76,61,48,18,13,28,48,59,17,5,20,0,14,10,28,15,14,26,11,24,24,4,7,6,57,78,61,104,67,108,93,158,119,99,67,73,93

	
```

```
		  

		9, Position,0,1000000,2000000,3000000,4000000,5000000,6000000,7000000,8000000,9000000,10000000,11000000,12000000,13000000,14000000,15000000,16000000,17000000,18000000,19000000,20000000,21000000,22000000,23000000,24000000,25000000,26000000,27000000,28000000,29000000,30000000,31000000,32000000,33000000,34000000,35000000,36000000,37000000,38000000,39000000,40000000,41000000
9,Count,73,158,86,80,111,111,116,111,69,170,41,67,85,48,16,3,18,9,7,22,7,4,5,2,11,17,10,21,14,52,5,49,49,42,94,162,107,110,77,87,78,26

	
```

```
		  

		10, Position,0,1000000,2000000,3000000,4000000,5000000,6000000,7000000,8000000,9000000,10000000,11000000,12000000,13000000,14000000,15000000,16000000,17000000,18000000,19000000,20000000,21000000,22000000,23000000,24000000,25000000,26000000,27000000,28000000,29000000,30000000,31000000,32000000,33000000,34000000,35000000,36000000,37000000
10,Count,9,31,8,18,54,17,26,50,19,5,5,0,7,5,61,44,47,46,7,11,4,43,48,131,134,102,125,115,111,137,116,122,123,141,181,113,113,67

	
```

```
		  

		11, Position,0,100000,200000,300000,400000,500000,600000,700000,800000,900000,1000000,1100000,1200000,1300000,1400000,1500000,1600000,1700000,1800000,1900000,2000000,2100000,2200000,2300000,2400000,2500000,2600000,2700000,2800000,2900000,3000000,3100000,3200000,3300000,3400000,3500000,3600000,3700000,3800000,3900000,4000000,4100000,4200000,4300000,4400000,4500000,4600000,4700000,4800000,4900000,5000000,5100000,5200000,5300000,5400000,5500000,5600000,5700000,5800000,5900000,6000000,6100000,6200000,6300000,6400000,6500000,6600000,6700000,6800000,6900000,7000000,7100000,7200000,7300000,7400000,7500000,7600000,7700000,7800000,7900000,8000000,8100000,8200000,8300000,8400000,8500000,8600000,8700000,8800000,8900000,9000000,9100000,9200000,9300000,9400000,9500000,9600000,9700000,9800000,9900000,10000000,10100000,10200000,10300000,10400000,10500000,10600000,10700000,10800000,10900000,11000000,11100000,11200000,11300000,11400000,11500000,11600000,11700000,11800000,11900000,12000000,12100000,12200000,12300000,12400000,12500000,12600000,12700000,12800000,12900000,13000000,13100000,13200000,13300000,13400000,13500000,13600000,13700000,13800000,13900000,14000000,14100000,14200000,14300000,14400000,14500000,14600000,14700000,14800000,14900000,15000000,15100000,15200000,15300000,15400000,15500000,15600000,15700000,15800000,15900000,16000000,16100000,16200000,16300000,16400000,16500000,16600000,16700000,16800000,16900000,17000000,17100000,17200000,17300000,17400000,17500000,17600000,17700000,17800000,17900000,18000000,18100000,18200000,18300000,18400000,18500000,18600000,18700000,18800000,18900000,19000000,19100000,19200000,19300000,19400000,19500000,19600000,19700000,19800000,19900000,20000000,20100000,20200000,20300000,20400000,20500000,20600000,20700000,20800000,20900000,21000000,21100000,21200000,21300000,21400000,21500000,21600000,21700000,21800000,21900000,22000000,22100000,22200000,22300000,22400000,22500000,22600000,22700000,22800000,22900000,23000000,23100000,23200000,23300000,23400000,23500000,23600000,23700000,23800000,23900000,24000000,24100000,24200000,24300000,24400000,24500000,24600000,24700000,24800000,24900000,25000000,25100000,25200000,25300000,25400000,25500000,25600000,25700000,25800000,25900000,26000000,26100000,26200000,26300000,26400000,26500000,26600000,26700000,26800000,26900000,27000000,27100000,27200000,27300000,27400000,27500000,27600000,27700000,27800000,27900000
11,Count,4,0,4,7,0,20,15,22,8,24,17,0,7,0,15,13,8,0,0,0,7,9,1,16,11,16,7,4,0,4,4,0,8,3,26,10,10,6,21,26,6,38,19,3,16,0,1,16,22,0,0,8,0,0,15,16,0,11,11,31,2,13,2,21,3,0,11,7,5,9,9,10,6,9,8,13,4,3,0,0,3,0,0,0,1,0,8,0,0,0,3,11,0,10,14,2,0,3,8,1,6,4,8,8,1,0,0,5,13,1,2,3,5,1,0,0,0,4,15,0,0,0,0,0,0,6,11,2,5,5,0,0,0,2,8,0,7,0,4,0,5,0,0,0,1,3,0,23,4,0,8,0,0,0,0,0,0,0,6,2,0,6,23,0,11,14,6,3,2,1,9,0,0,3,0,2,7,13,0,0,0,4,0,0,0,0,2,14,0,4,0,1,0,1,2,8,1,0,0,0,22,1,2,20,7,7,9,2,3,6,7,21,10,17,11,0,6,15,11,7,11,14,26,33,13,8,6,0,3,19,11,27,3,8,0,11,10,1,10,7,10,0,15,35,6,16,6,12,9,24,19,19,28,23,14,21,29,25,37,25,17,1,6,9,10,61,3,4,16,14,10,39,5,7,0,13,8,15,5,0

	
```

```
		  

		Un_random, Position,0,1000000,2000000,3000000,4000000,5000000,6000000,7000000,8000000,9000000,10000000,11000000,12000000,13000000,14000000,15000000,16000000,17000000,18000000,19000000,20000000,21000000,22000000,23000000,24000000,25000000,26000000,27000000,28000000,29000000,30000000,31000000,32000000,33000000,34000000,35000000,36000000,37000000,38000000,39000000,40000000,41000000,42000000,43000000,44000000,45000000,46000000
Un_random,Count,14,8,8,26,7,6,12,4,10,9,7,8,4,9,0,7,10,5,7,0,15,6,3,15,10,2,4,14,5,8,60,13,14,10,14,7,5,6,5,16,1,13,20,19,10,0,0

	
```


---

 **Details by gene** 

**Here** you can find a tab-separated table.
