# Supplementary material for: Association genetics of bunch weight and its component traits in East African highland banana (Musa spp. AAA group)
Source: Theor Appl Genet. 2019 Sep 16;132(12):3295–308. doi: 10.1007/s00122-019-03425-x (PMC6820618; doi:10.1007/s00122-019-03425-x)
Supplement: Supplementary file 2 — Supplementary material 2 (PDF 455 kb) [file 122_2019_3425_MOESM2_ESM.pdf]

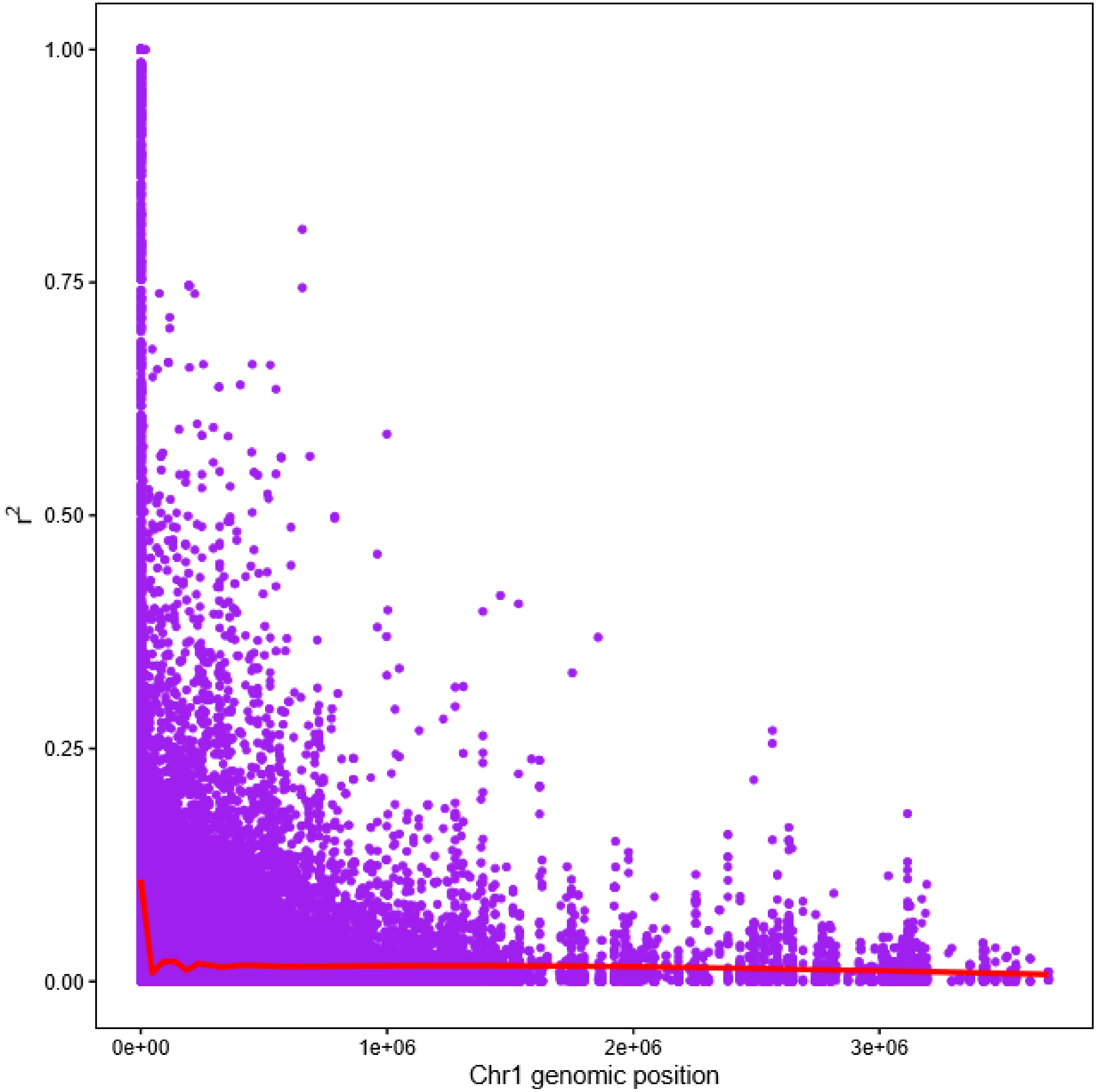

1.00
0.75
0.50
0.25
0.00
r^2
0e+00
1e+06
2e+06
3e+06
Chr1 genomic position

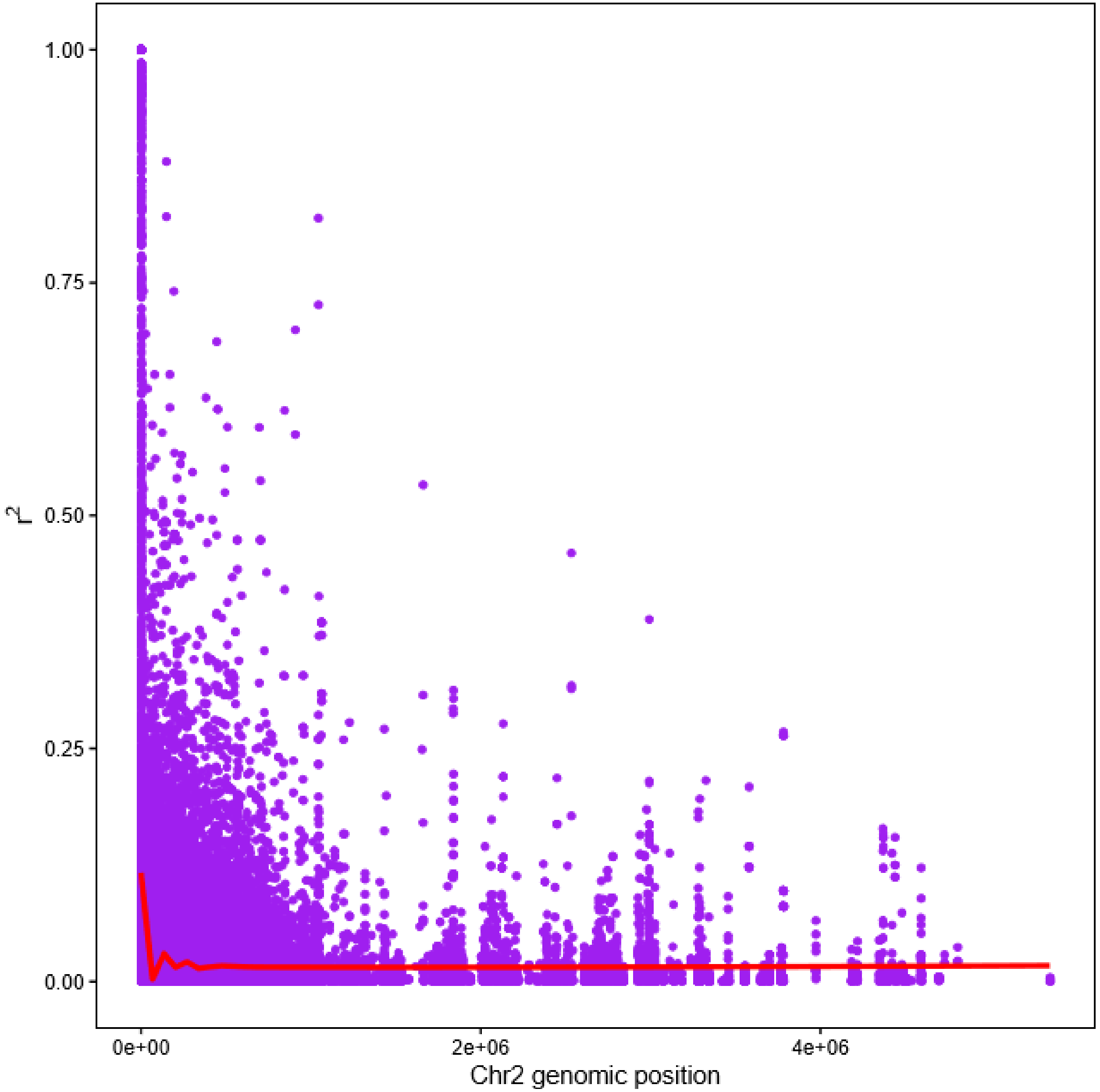

1.00
0.75
0.50
0.25
0.00
r^2
0e+00
2e+06
4e+06
Chr2 genomic position

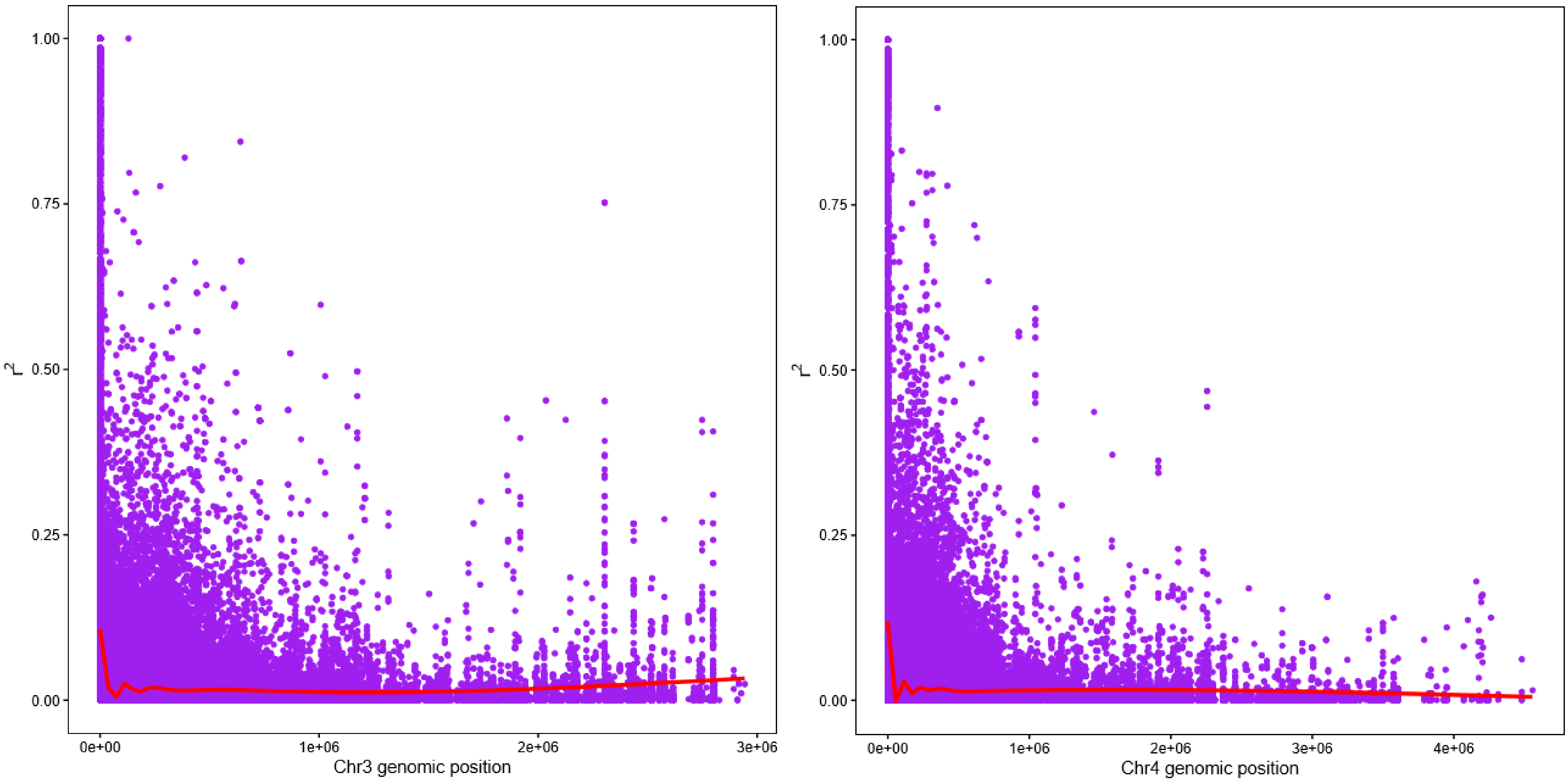

1.00
0.75
0.50
0.25
0.00
r2
0e+00
1e+06
2e+06
3e+06
Chr3 genomic position
1.00
0.75
0.50
0.25
0.00
r2
0e+00
1e+06
2e+06
3e+06
4e+06
Chr4 genomic position

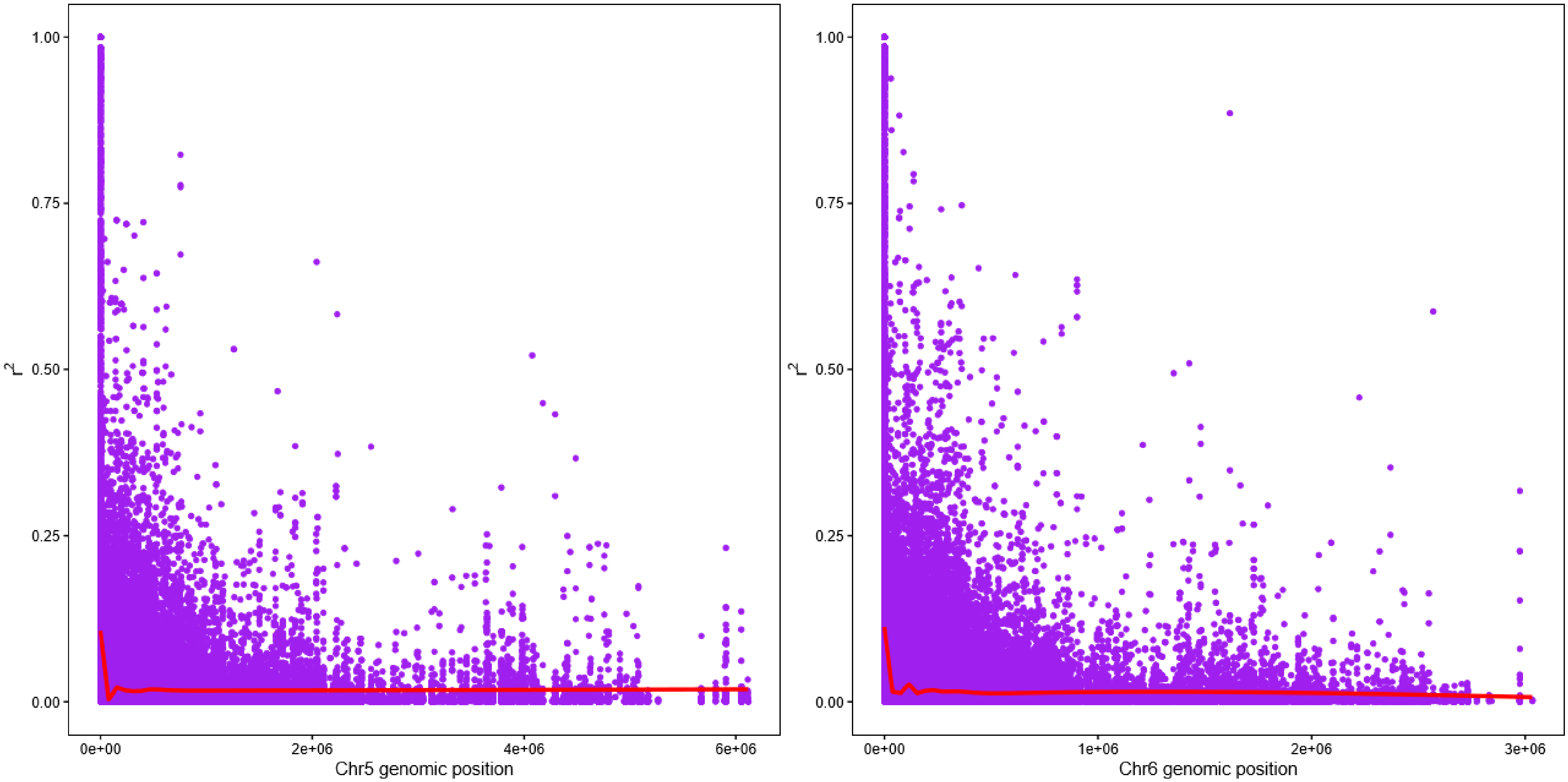
1.00
0.75
0.50
0.25
0.00
r2
0e+00
2e+06
4e+06
6e+06
Chr5 genomic position
1.00
0.75
0.50
0.25
0.00
r2
0e+00
1e+06
2e+06
3e+06
Chr6 genomic position

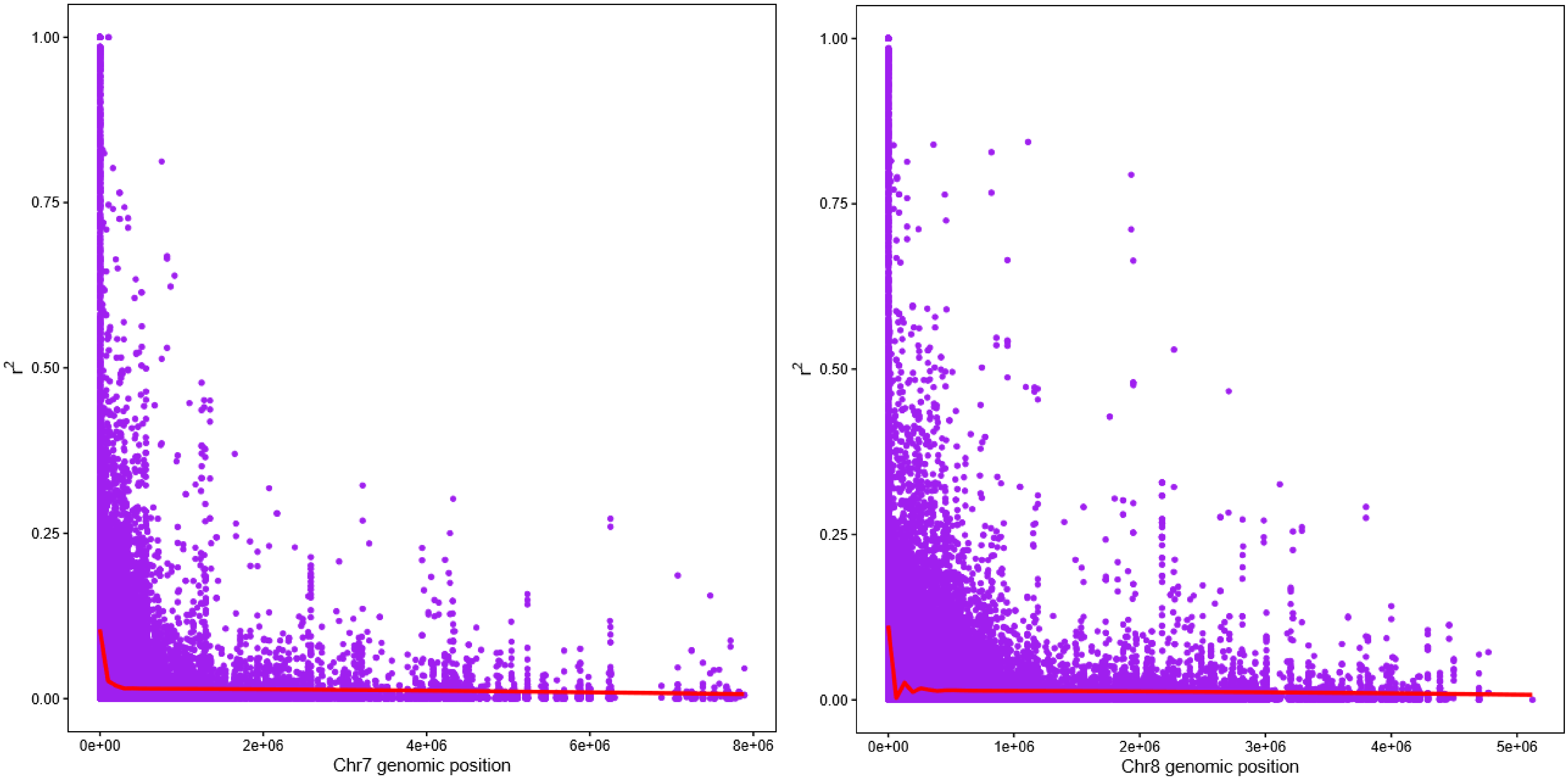
1.00
0.75
0.50
0.25
0.00
r^2
0e+00
2e+06
4e+06
6e+06
8e+06
Chr7 genomic position
1.00
0.75
0.50
0.25
0.00
r^2
0e+00
1e+06
2e+06
3e+06
4e+06
5e+06
Chr8 genomic position

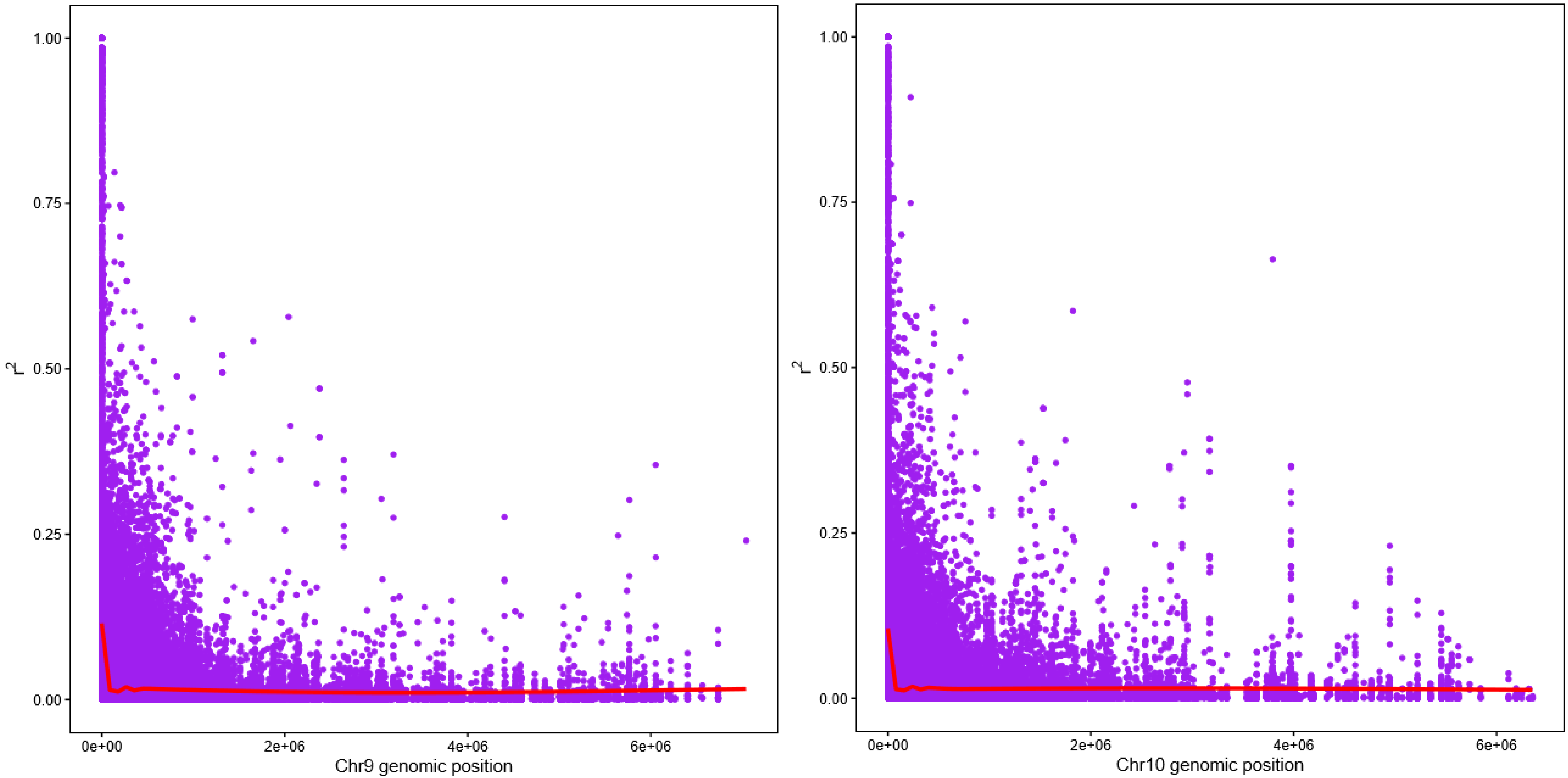
1.00
0.75
0.50
0.25
0.00
r2
0e+00
2e+06
4e+06
6e+06
Chr9 genomic position
1.00
0.75
0.50
0.25
0.00
r2
0e+00
2e+06
4e+06
6e+06
Chr10 genomic position

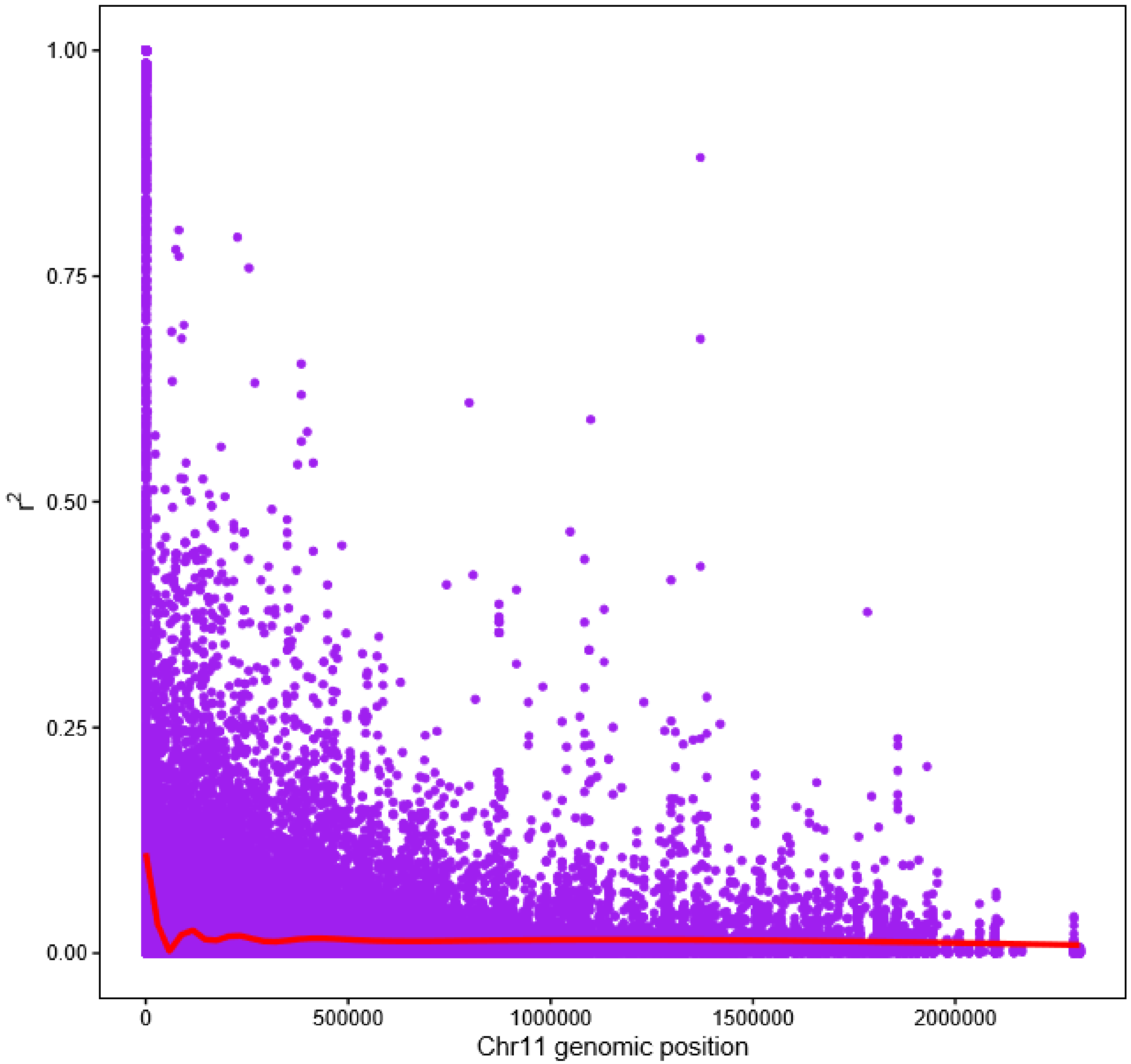

Fig S1. Linkage disequilibrium decay per chromosome. The smooth curve is based on generalized additive model (GAM) implimented by geom_smooth in ggplot R-package
