# Supplementary figures and images for: Association genetics of bunch weight and its component traits in East African highland banana (Musa spp. AAA group)
Source: Theor Appl Genet. 2019 Sep 16;132(12):3295–308. doi: 10.1007/s00122-019-03425-x (PMC6820618; doi:10.1007/s00122-019-03425-x)

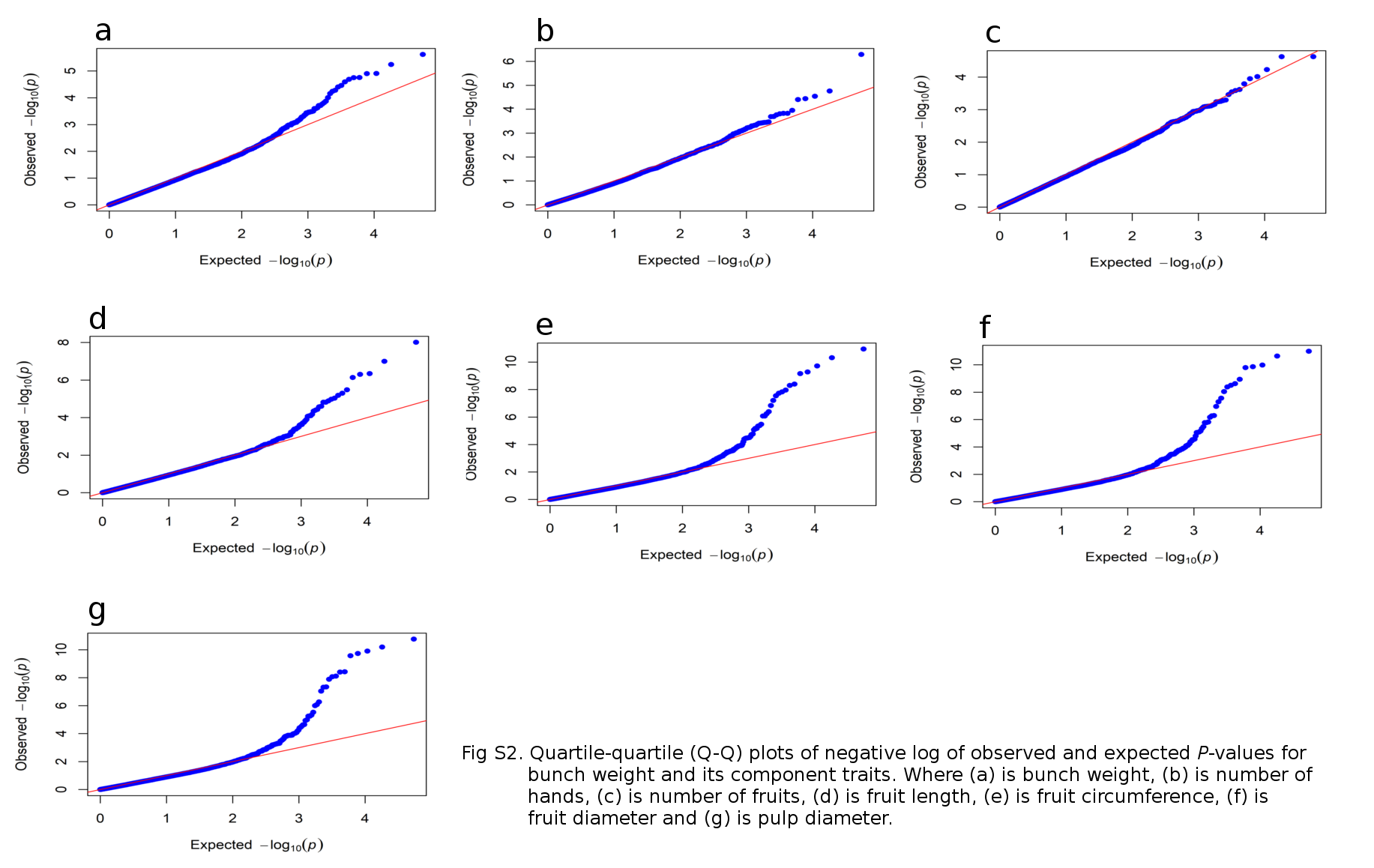

Supplement: Supplementary file 3 — Supplementary material 3 (TIFF 288 kb) [file 122_2019_3425_MOESM3_ESM.tiff]

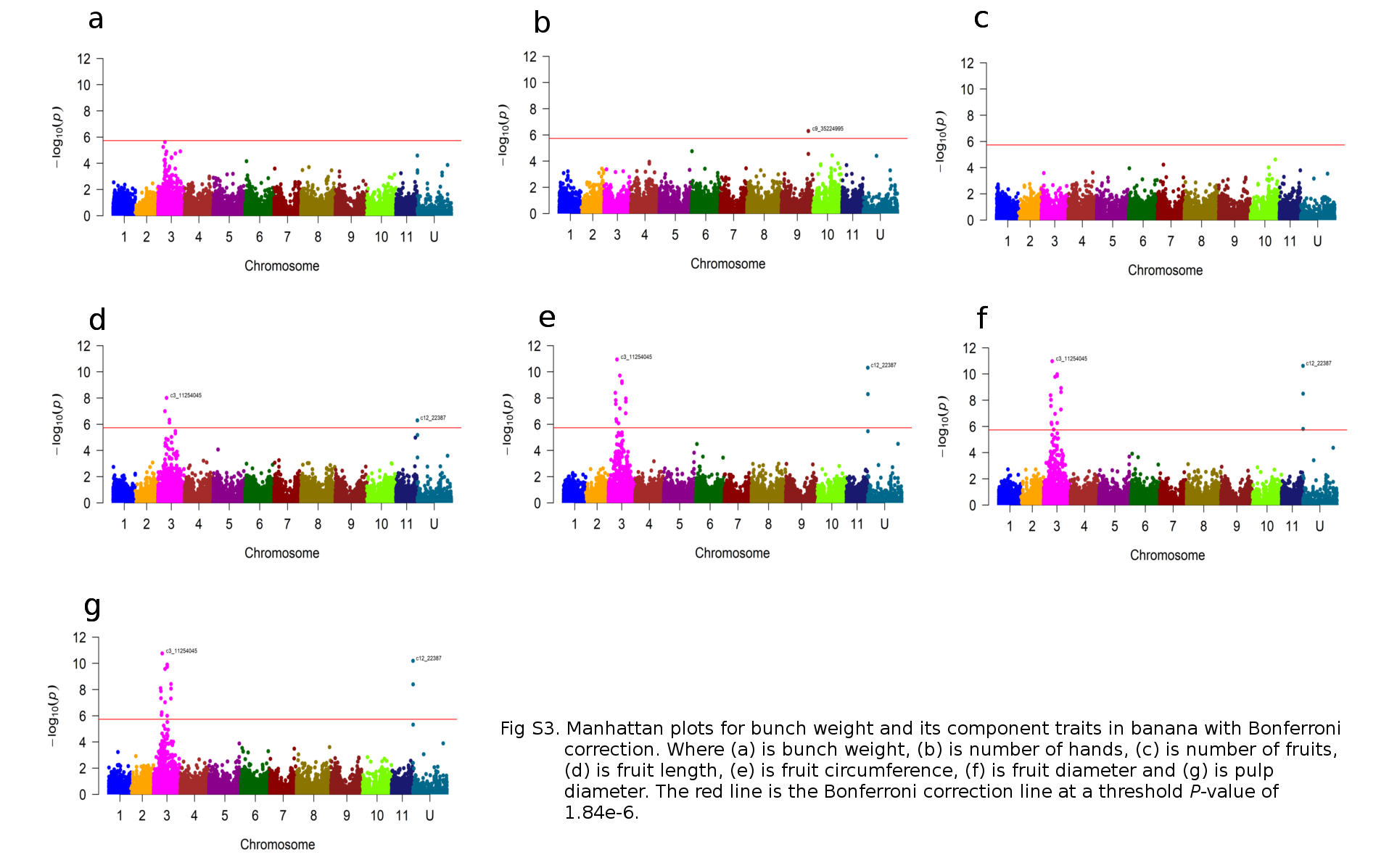

Supplement: Supplementary file 4 — Supplementary material 4 (TIFF 435 kb) [file 122_2019_3425_MOESM4_ESM.tiff]

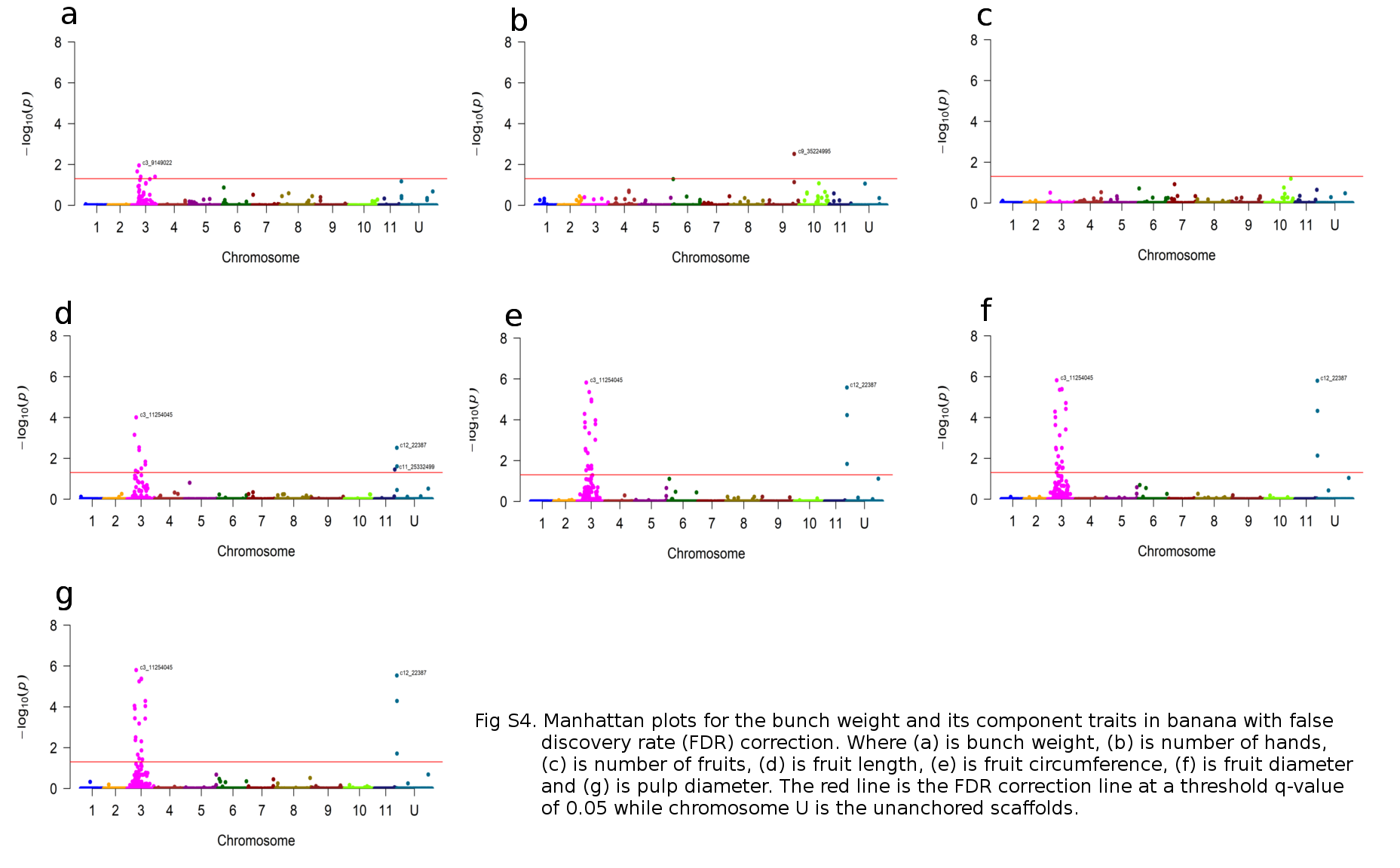

Supplement: Supplementary file 5 — Supplementary material 5 (TIFF 277 kb) [file 122_2019_3425_MOESM5_ESM.tiff]

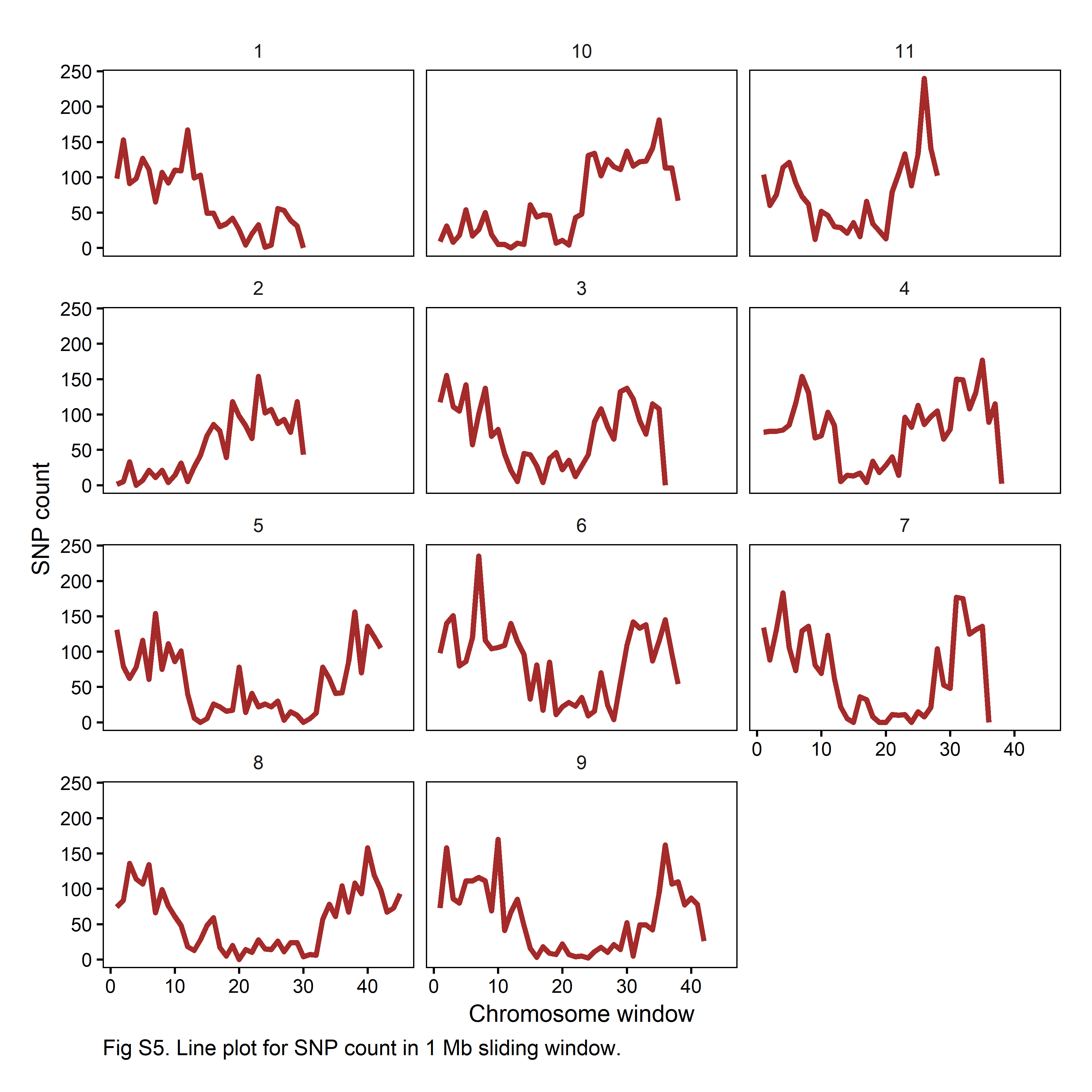

Supplement: Supplementary file 6 — Supplementary material 6 (TIFF 211 kb) [file 122_2019_3425_MOESM6_ESM.tiff]
